# Multimodal profiling of the transcriptional regulatory landscape of the developing mouse cortex identifies Neurog2 as a key epigenome remodeler

**DOI:** 10.1038/s41593-021-01002-4

**Published:** 2022-02-07

**Authors:** Florian Noack, Silvia Vangelisti, Gerald Raffl, Madalena Carido, Jeisimhan Diwakar, Faye Chong, Boyan Bonev

**Affiliations:** 1 Helmholtz Pioneer Campus, Helmholtz Zentrum München, Neuherberg, Germany; 2 Physiological Genomics, Biomedical Center, Ludwig-Maximilians-Universität München, Munich, Germany

**Keywords:** Developmental neurogenesis, Epigenomics, Gene regulatory networks, Gene regulation

## Abstract

How multiple epigenetic layers and transcription factors (TFs) interact to facilitate brain development is largely unknown. Here, to systematically map the regulatory landscape of neural differentiation in the mouse neocortex, we profiled gene expression and chromatin accessibility in single cells and integrated these data with measurements of enhancer activity, DNA methylation and three-dimensional genome architecture in purified cell populations. This allowed us to identify thousands of new enhancers, their predicted target genes and the temporal relationships between enhancer activation, epigenome remodeling and gene expression. We characterize specific neuronal transcription factors associated with extensive and frequently coordinated changes across multiple epigenetic modalities. In addition, we functionally demonstrate a new role for Neurog2 in directly mediating enhancer activity, DNA demethylation, increasing chromatin accessibility and facilitating chromatin looping in vivo. Our work provides a global view of the gene regulatory logic of lineage specification in the cerebral cortex.

## Abstract (web summary)

By profiling multiple epigenetic layers and enhancer activity in vivo, the authors show a widespread remodeling of the regulatory landscape during mouse cortical development and identify Neurog2 as a key transcription factor driving this process.

## Main

During development, cellular identity is established by the complex interplay between transcriptional regulators, *cis*-regulatory elements (CREs) and the chromatin landscape, taking place within the physical constraints imposed by three-dimensional (3D) nuclear architecture^1,2^. These different layers of molecular interactions form the basis of gene regulatory networks (GRNs), ensuring the precise temporal and spatial regulation of gene expression. Although our understanding of the underlying molecular cascades has grown considerably, the exact multilayered mechanisms leading to acquisition of neural identity, lineage specification and developmental plasticity in the cerebral cortex remain unclear.

Recently, profiling the transcriptional and chromatin accessibility landscape at single-cell resolution has improved our understanding of the temporal logic of lineage specification in the mouse^3^ and human^4,5^ developing cerebral cortex. However, studies that comprehensively assess the effect of multiple regulatory modalities on transcription remain scarce^6–9^. Many of these regulatory layers converge on CREs such as enhancers, which represent the key building blocks of GRNs in eukaryotes. Changes in histone modifications, accessibility and the binding of cell-type-specific transcription factors (TFs) have been proposed to explain the relationship between enhancer activation and gene expression^10,11^. Using 3D proximity data improves target gene prediction^12^, yet such data is rarely cell-type specific. Furthermore, some enhancers have been shown to interact only weakly or not at all with their target promoters, therefore challenging the classical view of stable enhancer–promoter (E–P) loops^13,14^. TF-associated chromatin looping has also been proposed to play a role in mouse^15^ and human^9^ brain development, yet the causal relationships between TF binding, epigenetic signature, enhancer activity, chromatin looping and tissue-specific gene expression remain to be understood.

To comprehensively assess how coordinated epigenome remodeling governs cell fate decisions in the developing neocortex in vivo, we integrated single-cell transcriptomic and chromatin accessibility data with cell-type-specific massive parallel reporter assay (MPRA), DNA methylation and 3D genome architecture. We identify thousands of new cell-type-specific enhancer–gene pairs (EGPs) and show that although enhancer activation appears to precede gene expression, only a subset of enhancers bound by specific TFs acts as truly lineage priming. In addition, we validate the cell-type-specific activity of the vast majority of these enhancers using a new MPRA approach in vivo and show that mutating specific TF motifs is sufficient to abolish reporter activity. Extending our analysis to DNA methylation and 3D genome topology, we show that despite overall cell-type specificity, regulatory loops vary considerably in both contact strength and dynamics. Finally, using integrated analysis and in vivo validation, we identify a new role for the TF Neurog2 in directly mediating enhancer activity, DNA demethylation, as well as leading to increased chromatin accessibility and chromatin looping in vivo. Thus, we propose that TFs represent a key component of the cell-type-specific reorganization of the chromatin landscape during lineage specification in the neocortex and can coordinate changes across multiple regulatory layers to facilitate lineage decisions. The generated data are freely available at https://shiny.bonevlab.com/ for interactive visualization.

## Results

### Single-cell transcription dynamics in the E14.5 mouse cortex

**Fig. 1:**
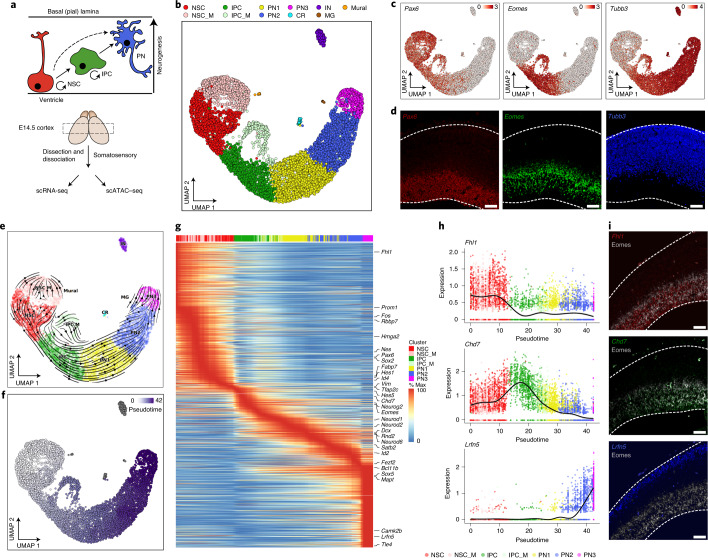
scRNA-seq analysis of mouse E14.5 cortical development. **a**, Schematic representation of the model system and the experimental approach. **b**, scRNA-seq UMAP projection. INs, interneurons; CRs, Cajal-Retzius neurons; MG, microglia. Mural represents mural cells. _M indicates the corresponding mitotic (G_2_-M) population. **c**, UMAP visualization with expression levels of the indicated marker genes. **d**, Representative immunofluorescence images of the indicated genes in coronal sections of E14.5 cortex. Scale bars, 50 µm. **e**, Direction of neuronal differentiation inferred from estimated RNA velocities and plotted as streamlines on the UMAP. **f**, Trajectory analysis depicting the inferred pseudotime on the UMAP projection. **g**, Pseudotime heat map ordering of the top 3,000 most variable genes across neural differentiation. **h**, Expression levels of the indicated genes across differentiation. Each dot shows the expression in an individual pseudotime-ordered cell, while the line represents the smoothed fit of expression levels. **i**, Representative FISH images of the depicted genes in coronal sections of E14.5 cortex. Scale bars, 50 µm.

To identify changes in the gene regulatory landscape upon differentiation at the single-cell level, we performed in parallel single-cell RNA sequencing (scRNA-seq) and single-cell assay for transposase-accessible chromatin using sequencing (scATAC–seq) from the mouse somatosensory cortex at midneurogenesis (embryonic day (E) 14.5; Fig. 1a and Methods).

**Extended Data Fig. 1:**
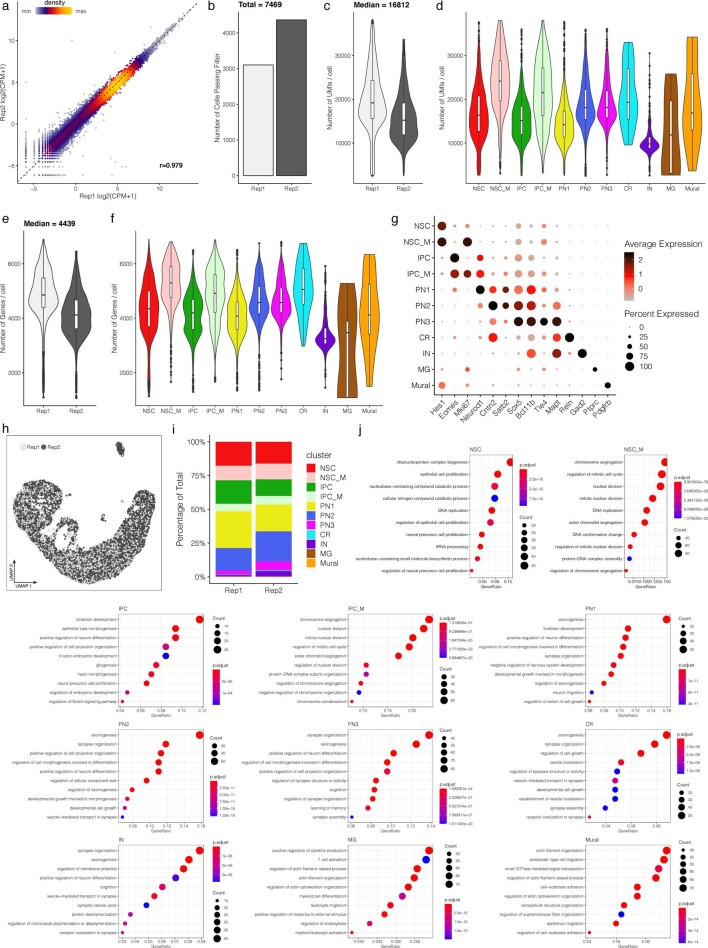
Quality control and further validation of the scRNA-seq data. **(a)** Scatterplot of gene expression values in the two biological scRNA-seq replicates, colored by density. The correlation (r) represents the Pearson correlation across all genes. **(b)** Barplot showing the number of cells passing quality filtering in each replicate. **(c-d)** Violin and box-whisker plots depicting the distribution of unique transcripts per cell across the two replicates and the identified clusters. **(e-f)** same as in **(c-d)** but showing the number of genes per cell. **(g)** Dot plot depicting the percentage of cells and gene expression levels of marker genes across the identified scRNA-seq clusters. **(h)** Overlay of replicate identity on the UMAP scRNA projection. **(i)** Stacked barplot showing the percentage of cells contributing to each cluster per replicate. **(j)** GOterm enrichment analysis of cluster specific marker genes.

**Extended Data Fig. 2:**
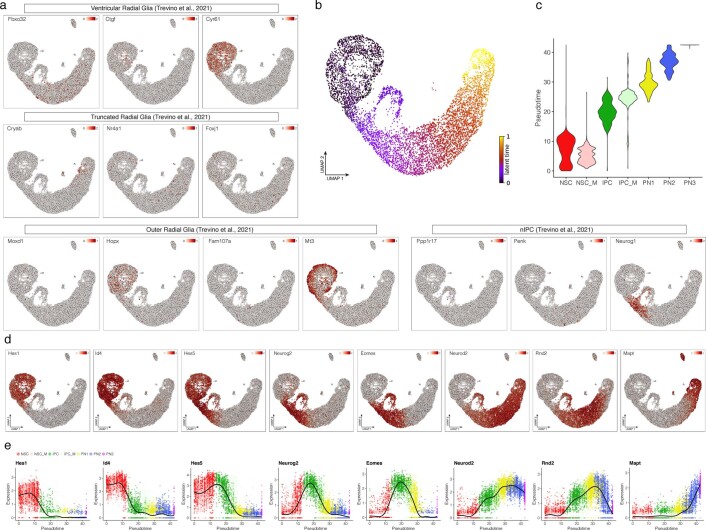
Pseudo-temporal analysis and comparison to human data. **(a)** UMAP visualization with expression levels of cell-type-specific marker genes identified in the human cortex^4^. **(b)** RNA velocity based inferred pseudotime depicted on the UMAP projection. Note the similarities with a trajectory-based approach (Fig. 1f) **(c)** Violin plots depicting the distribution of pseudotime values per cluster. **(d)** UMAP visualization with expression levels of the indicated genes. **(e)** Gene expression changes along differentiation pseudotime. Each dot shows the expression per cell of the indicated gene, the line represents the smoothed fit of expression levels along pseudotime.

The scRNA data were of high quality and were highly reproducible across replicates (Extended Data Fig. 1a–f and Supplementary Data 1). We identified 11 clusters that recapitulated the major cell types of the developing cortex (Fig. 1b–d and Extended Data Fig. 1d–i). Progenitor populations such as neural stem cells (NSCs) and intermediate progenitor cells (IPCs) were characterized by Gene Ontology (GO) terms such as proliferation and Notch signaling, while projection neurons (PNs; 1–3) were associated with axonogenesis, synapse formation and cognition (Extended Data Fig. 1j). We then examined markers for different types of human radial glia cells, whose existence in the mouse is still under debate^16^. We found that, in comparison to human fetal cortex data^4^, markers for both ventricular and outer radial glia cells were nonoverlapping in the mouse (Extended Data Fig. 2a), suggesting differences in how these genes are regulated.

To infer the developmental trajectory and identify changes in gene expression, we used a generalized RNA velocity approach^17^ (Fig. 1e and Extended Data Fig. 2b) and Monocle3 (ref. ^18^; Fig. 1f,g and Extended Data Fig. 2c). Consistent with previous lineage-tracing results^19^, our trajectory analysis revealed transcriptional waves of key neurogenic factors in NSCs (*Hes1*, *Id4* and *Hes5*), IPCs (*Neurog2* and *Eomes*), PN1 (*Neurod2*), PN2 (*Rnd2*) and PN3 (*Mapt*) (Extended Data Fig. 2d,e and Supplementary Data 2). In addition, we identified a number of genes exhibiting variable expressions along the studied differentiation trajectory, which we verified by independent fluorescence in situ hybridization (FISH) experiments (Fig. 1g–i). Such genes include *Fhl1* (involved in muscular dystrophy^20^), *Chd7* (a member of the chromodomain family of chromatin remodelers and associated with the CHARGE syndrome^21^) and the cell adhesion protein *Lrfn5*, which has been linked to autism, and mental retardation^22^.

Collectively, our scRNA-seq data recapitulate known transcriptional dynamics during neurogenesis, reveal cell-type-specific expression and provide a molecular roadmap to investigate the influence of epigenetic regulation on gene expression in the context of corticogenesis.

### Single-cell chromatin accessibility and transcription factor binding motifs

**Extended Data Fig. 3:**
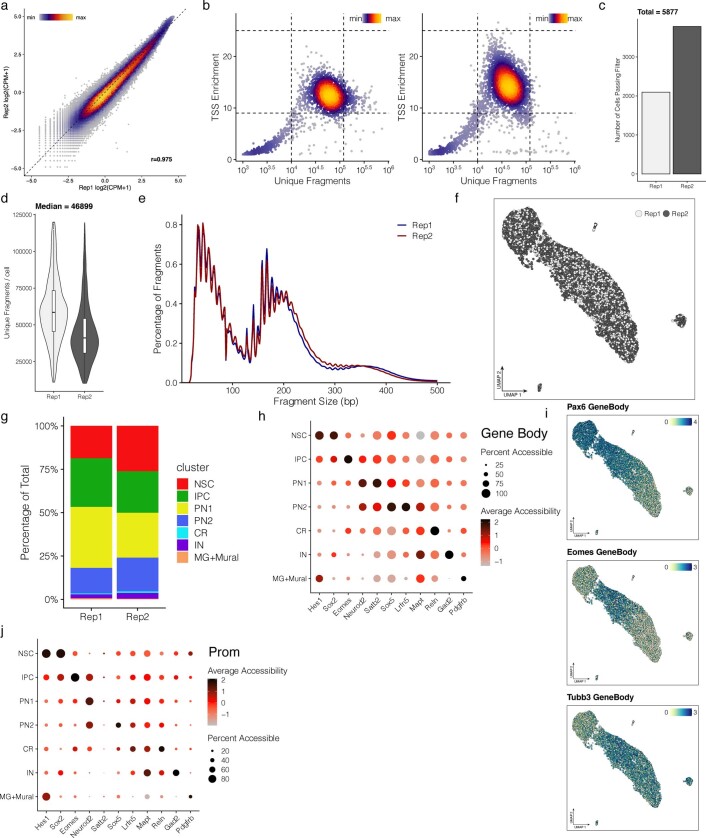
Quality control and validation of the scATAC-seq data. **(a)** Scatterplot of the aggregated accessibility values in the two biological scATAC-seq replicates, colored by density. The correlation (r) represents the Pearson correlation across all 370975 peaks. **(b)** Scatter plot of the quality control metrics (TSS enrichment and unique fragments per cell) per replicate, colored by density. Dashed lines represent the filters for high-quality cells, retained for further analysis. **(c)** Barplot showing the number of cells passing quality filtering in each replicate. **(d)** Violin and box-whisker plots depicting the distribution of unique fragments per cell across the two replicates. **(e)** Aggregated scATAC-seq fragment size distribution across replicates demonstrating sub-, mono- and multi-nucleosome spanning fragments. **(f)** Overlay of the replicate identity on the UMAP scATAC projection. **(g)** Stacked barplot showing the percentage of cells contributing to each cluster per replicate. **(h)** Dot plot depicting the percentage of cells and the average gene body accessibility of marker genes across the identified scATAC-seq clusters. **(i)** UMAP visualization with gene body accessibility of the indicated genes **(j)** Same as in **(h)** but based on promoter accessibility.

To dissect how transcriptional dynamics are associated with the remodeling of the epigenome landscape, we next focused on chromatin accessibility. Similar to the scRNA-seq data, the scATAC–seq data were characterized by high quality and strong correlation across replicates (Extended Data Fig. 3a–e and Supplementary Data 1 and 3).

**Fig. 2:**
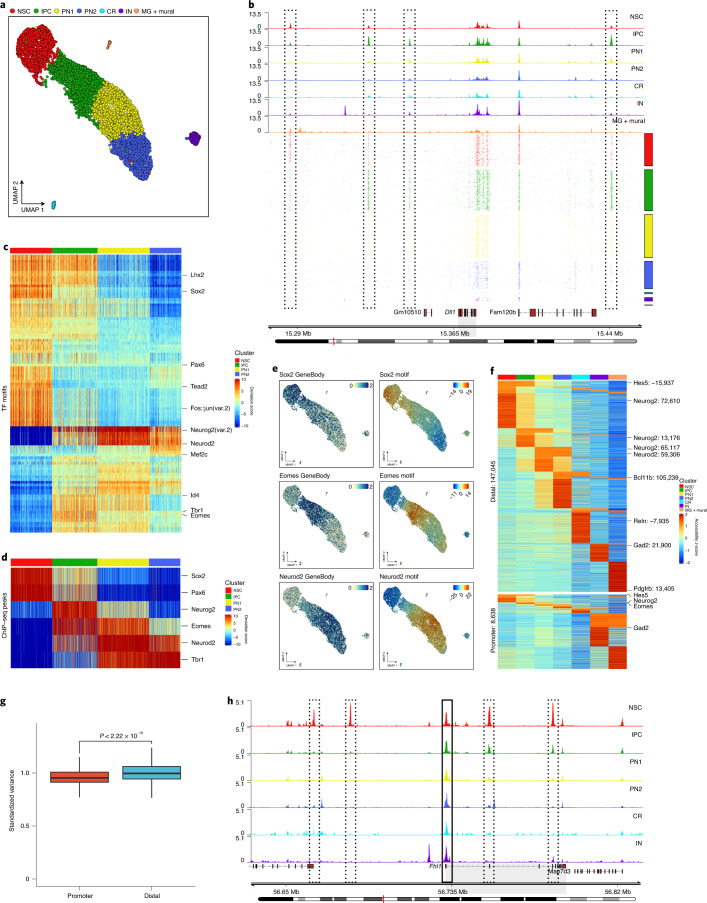
scATAC–seq identifies dynamic transcription factor motifs and variable distal regulatory elements. **a**, scATAC–seq UMAP projection (cells derived from the same brain as the scRNA-seq data). **b**, Genomic tracks showing the accessibility of aggregated scATAC-seq clusters (top) and of 1,000 random single cells (bottom) at the *Dll1* gene locus. Four examples of differential accessible loci are highlighted with dashed black rectangles. **c**,**d**, Heat map clustering of chromVAR bias-corrected accessibility deviations for the most 100 variably expressed TFs (**c**) or based on ChIP–seq peaks (**d**). **e**, Gene body accessibility or chromVAR motif bias-corrected deviations of the indicated TFs. **f**, Heat map of aggregated accessibility *z*-scores (per cluster) of differentially accessible peaks in distal regions or within promoters. Labels indicate the name and the distance in base pairs (bp) to the nearest TSS. **g**, Box plot showing the standardized variance within promoter (*n* = 81,810) or distal (*n* = 289,165) regions. Plot displays the median (line), 25th and 75th percentiles (box), as well as the 10th and 90th percentiles (whiskers). Statistical significance was calculated using a two-sided Wilcoxon rank-sum test. **h**, Genomic tracks showing scATAC accessibility at the *Fhl1* gene locus (highlighted in gray). The promoter region (solid black rectangle) and four putative regulatory elements (dashed black rectangles) are also highlighted.

We identified seven highly reproducible clusters (Fig. 2a and Extended Data Fig. 3f,g), which we subsequently annotated based on the gene body accessibility of known marker genes (Extended Data Fig. 3h,i). Similarly to the scRNA-seq, we observed a gradual progression in cell state (Fig. 2a). However, no distinct mitotic clusters were identified, presumably due to the high similarities of the chromatin accessibility landscape during different cell cycle phases^4,23^. As an example, we observed chromatin accessibility changes at the *Dll1* locus even at the single-cell level (Fig. 2b).

To identify TFs whose binding correlates with accessibility changes, we used ChromVAR (Fig. 2c and Supplementary Data 4)^24^. NSC-specific factors included known neural TFs such as Sox2, Pax6 or Lhx2, as well as the less-characterized Tead2 and c-Fos/Jun (AP-1), which have been recently described as important in NSCs^25,26^. Conversely, neurogenic TFs such as Eomes and Neurod2 had increased motif accessibility during the transition from NSC to IPC and IPC to PN1, respectively (Fig. 2c–e).

Most changes in chromatin accessibility occurred at CREs rather than on promoters (Fig. 2b,f–h, Extended Data Fig. 3j and Supplementary Data 5), consistent with the hypothesis that promoter accessibility, while required, may not be sufficient for transcription^27^.

Together, our scATAC–seq data identify major reorganization of the chromatin landscape during neural differentiation at the single-cell level, associated with cell-type-specific TFs. We also demonstrate that accessibility at CREs is highly dynamic and frequently correlated with changes in gene expression, compared to the relatively invariant/static promoters.

### Dynamic enhancer–gene pairs underlie neuronal commitment

**Extended Data Fig. 4:**
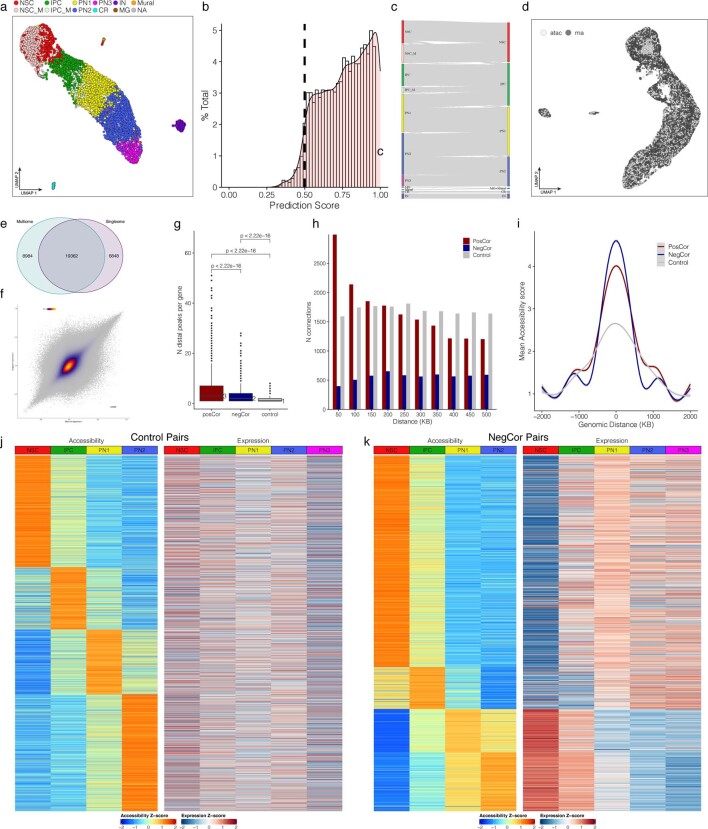
scATAC-scRNA integration metrics and identification of enhancer-gene pairs. **(a)** scATAC UMAP projection with labels transferred from scRNA-seq. **(b)** Histogram-density plot depicting the distribution of prediction scores obtained during the scRNA-seq to scATAC-seq label transfer. Only cells with scores above the cutoff (dashed line) were retained. **(c)** Sankey plot showing the correspondence between scRNA-based labels (left) to scATAC-based cluster identities (right). **(d)** UMAP projection of the integrated scRNA-scATAC dataset, colored by method. **(e)** Venn diagram displaying the overlap between enhancer gene pairs identified using either multiome or computational integrate singleome data (74%). **(f)** Scatterplot depicting the linkage scores of enhancer-gene pairs identified using the two complementary methods (computational vs multiome) (Pearson’s r=0.63). **(g)** Box-plot depicting the number of distal linked peaks per gene (n=3246 posCor, 1675 negCor and 10163 control genes). Statistical significance is calculated using a two-sided wilcoxon rank-sum test. Plot displays median with corresponding value on the side (line), 25^th^ or 75^th^ percentiles (box), 10^th^ or 90^th^ percentiles (whisker) as well as outliers (dots). **(h)** Histogram displaying the genomic distance between TSS and positively, negatively or non-correlated (Control) peak-gene pairs. **(i)** Aggregated average accessibility plot (± 2kb) of the three categories of peak-gene pairs. **(j-k)** Heatmaps of aggregated accessibility of distal elements and gene expression levels of their linked genes for each of the identified non-correlated (Control) and negatively correlated pairs (NegCor). Rows were clustered by enhancer accessibility using feature binarization (Methods).

To uncover how dynamic accessibility at CREs relates to changes in gene expression, we integrated the scRNA-seq and scATAC–seq data, which resulted in overall high prediction scores, minimal cross-annotations and high intermixed projection on a joint uniform manifold and approximation projection (UMAP; Extended Data Fig. 4a–d). To identify EGPs, we used a correlation-based analysis^28^ (Methods), which we validated using publicly available single-cell multiome data (Extended Data Fig. 4e,f). We identified 16,978 positively correlated EGPs (Supplementary Data 6; *r* ≥ 0.35, false discovery rate (FDR) ≤ 0.1) with each gene being connected to a median of three distal regions (Extended Data Fig. 4g). Positively correlated distal regions were usually located closer to their predicted target genes (Extended Data Fig. 4h) and were characterized by overall higher accessibility compared to non-correlated pairs (also referred to as control pairs; −0.35 ≥ *r* ≤ 0.35, FDR > 0.1) but not compared to negatively correlated pairs (*r* ≤ −0.35, FDR ≤ 0.1; Extended Data Fig. 4i).

**Fig. 3:**
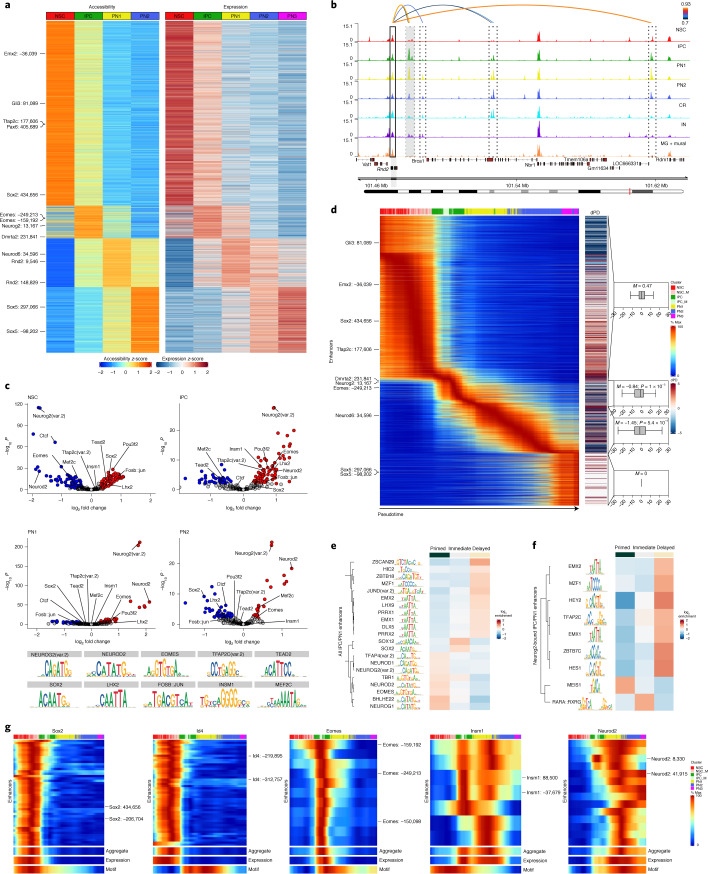
Lineage dynamics of enhancer–gene pairs and transcription factor motifs. **a**, Heat maps of aggregated accessibility of putative enhancers (left) and gene expression levels of their linked genes (right) for each of the 16,978 positively correlated EGPs (rows). Rows were clustered by enhancer accessibility using feature binarization (Methods). **b**, Genomic tracks depicting aggregated accessibility (per cluster) at the *Rnd2* gene locus. Arcs represent Rnd2 linked enhancers and their correlation score. The promoter region (solid black rectangle), previously characterized enhancers (gray dashed rectangle) and predicted enhancers (black dashed rectangles) are highlighted. **c**, Scatterplot showing the enrichment of TF motifs within cluster-specific EGPs. Red and blue dots indicate significantly (log_10_*P* ≥ 2; abs(log fold change) ≥ 0.25) enriched and depleted motifs, respectively. The significance of each motif was calculated using Fisher’s exact test. **d**, Left: heat map depicting scaled enhancer accessibility of EGPs in individual cells ordered along the integrated pseudotime. Right: heat map depicting the pseudotemporal difference between maximum enhancer accessibility and expression of the linked gene (referred to as ‘dPD’), as well as box plots with the median of these differences (*M*). Negative values mean that accessibility precedes gene expression. Box plots displays the median (line), 25th and 75th percentiles (box limits) as well as the 10th and 90th percentiles (whiskers). Significance was calculated using a one-sample, one-sided Wilcoxon signed-rank test. **e**, Heat map showing motif enrichment of primed (dPD ≤ −2; dark green), immediate (dPD > −2 and <2; gray) and delayed (dPD ≥ 2) positive correlated enhancers. Only motifs of expressed TFs are displayed. **f**, Same as **e**, but only for enhancers bound by Neurog2 and containing its binding motif. **g**, Pseudotime heat map ordering of enhancer accessibility (individual or aggregate), linked gene expression and motif accessibility of the indicated TFs.

To address if the identified EGPs are cell-type specific, we clustered the distal regions based on their pseudobulk accessibility. We found that only the positively correlated EGPs were mostly cell-type specific (Fig. 3a and Extended Data Fig. 4j,k). To further validate these findings, we focused on the *Rnd2* locus (a gene involved in neuronal migration^29^), which was upregulated in IPCs and mainly expressed in newborn neurons (PN1; Extended Data Fig. 2d,e). We identified multiple EGPs associated with *Rnd2* (Fig. 3b), including a previously validated enhancer^29^.

**Extended Data Fig. 5:**
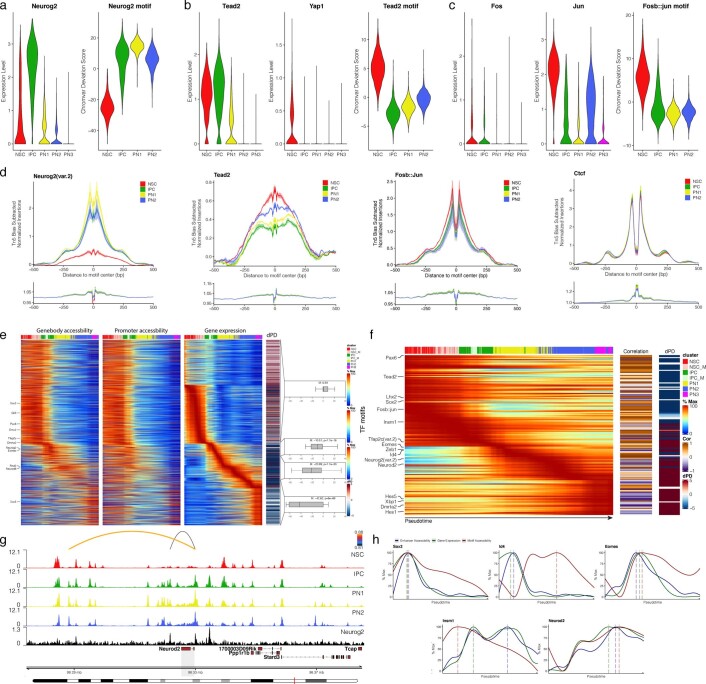
Properties of the identified enhancer-gene links. **(a-c)** Violin plots depicting gene expression levels (left) and deviation in accessibility between clusters (right) of the indicated transcription factors and their corresponding binding motifs. **(d)** Motif footprint of different transcription factors (with Tn5 insertion bias) in the indicated scATAC clusters. **(e)** Left heatmaps depict scaled gene body as well as promoter accessibility and gene expression levels of enhancer-gene pairs in individual cells ordered along the same integrated pseudotime as in Fig. 3d. Outmost right heatmap depicting the pseudo-temporal difference between maximum promoter accessibility and expression of the linked gene (referred to as ‘dPD’), as well as box-whisker plots with the median of these differences (M). Negative values mean that promoter accessibility precedes gene expression. Significance was calculated using one-sample Wilcoxon signed rank test. **(f)** Heatmaps depict the pseudotemporal ordering of chromVAR scaled deviation scores (left), the correlation between motif accessibility and TF expression (middle) and difference between maximum motif accessibility and TF expression (referred to as ‘dPD’). **(g)** Genomic tracks depicting the aggregated accessibility (per cluster) at the Neurod2 gene locus with arcs on top representing the identified enhancer-gene pairs, colored by Pearson correlation of the enhancer accessibility and gene expression. Neurog2 ChIPseq track is also depicted (black). **(h)** Smoothed fit line plots depicting the scaled aggregated enhancer accessibility, expression of the linked transcription factors and its motif accessibility across pseudotime. Dashed lines indicate the pseudo-temporal maxima.

To address which TFs are associated with the identified cell-type-specific enhancers, we used motif enrichment (Methods). We found that some NSC-specific TFs such as Sox2, Tead2 and AP-1 (Fos::jun) were enriched only in NSC-specific enhancers, while others such as Lhx2 and Pou3f2 (also known as Brn2) were present in both NSC and IPC enhancers (Fig. 3c). Conversely, neurogenic TFs such as Neurog2, Eomes and Neurod2 were depleted in NSC enhancers but strongly enriched in both IPC and PN1/2. Comparing expression patterns with motif footprints corroborated these findings (Extended Data Fig. 5a–d).

To determine the temporal relationship between chromatin accessibility and gene expression, we ordered the EGPs based on their enhancer accessibility as a function of the integrated pseudotime (Fig. 3d, Extended Data Fig. 5e and Supplementary Data 7). This systematic analysis showed that for transient cell states (IPC, PN1 and PN2) enhancer accessibility generally precedes upregulation of their linked gene. Expression and downstream motif accessibility of transcriptional activators such as Pax6, Sox2, Eomes and Neurog2 were highly correlated, while known repressors such as Insm1, Id4 and Hes1 displayed a strong negative correlation (Extended Data Fig. 5f).

Next, we examined if all predicted enhancers of a gene are characterized by similar accessibility dynamics. While most became accessible shortly before or at the onset of gene expression (Fig. 3g and Extended Data Fig. 5h), some (such as Eomes: −159,192 and Neurod2: 8,330) acquired accessibility considerably earlier, suggesting that they may act in lineage priming and be bound by specific TFs such as Neurog2 at the Neurod2 primed enhancer (Extended Data Fig. 5g). Consistent with this hypothesis, we found that motifs for neural bHLH (Neurog and Neurod) and T-box (Eomes and Tbr1) TFs were enriched in primed enhancers, while Lhx and Emx motif families were present in enhancers induced with or after the onset of expression (Fig. 3e). Surprisingly, Meis1 motifs were enriched in Neurog2-bound primed enhancers, while others (Emx and Tfap2) were present in delayed enhancers (Fig. 3f). These findings suggest that the priming activity of pioneer TFs might be regulated by cofactors such as Meis1 or repressors such as Hes/Hey1.

In summary, integrating scRNA-seq and scATAC–seq allowed us to identify EGPs, which led to several new findings. First, we find specific TFs enriched at cell-type-specific enhancers. Second, we show that only a subset of enhancers appears to be lineage priming and that cofactors/repressors may influence TF pioneering activity. Finally, the identification of EGPs in single cells, in vivo, sets the stage to systematically interrogate how TFs facilitate and maintain GRNs during development.

### In vivo MPRA uncovers cell-type-specific enhancer activity

**Fig. 4:**
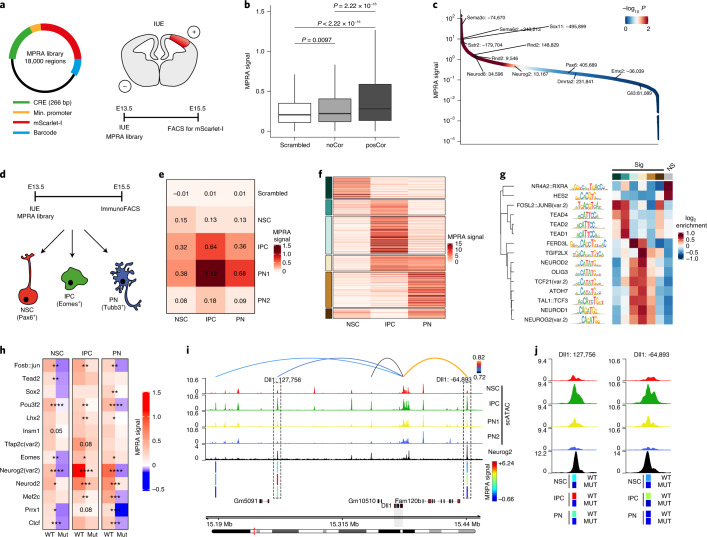
In vivo immunoMPRA validates cell-type-specific activity of identified CREs and their regulation by transcription factors. **a**, Experimental overview of the in vivo MPRA. **b**, Box plots depicting MPRA activity for scrambled control (white; *n* = 1,713) and CREs of non-correlated (noCor; *n* = 873) or positively correlated (posCor; *n* = 7,772) EGPs. Plot displays the median (line), 25th and 75th percentiles (box limits) as well as the 10th and 90th percentiles (whiskers). Statistical significance was calculated using a two-sided Wilcoxon rank-sum test. **c**, Ranked plot showing MPRA signal of individual CREs from posCor EGPs. Individual points are ordered and color-coded by the significance of their activation compared with scrambled controls. **d**, Experimental overview of the in vivo immunoMPRA approach. **e**, Heat map displays the cell-type-specific median MPRA signal from scrambled controls and of CREs from positively correlated EGPs. **f**, Partition around medoids clustering of significantly active CREs from positively correlated EGPs. Each row represents a single CRE. **g**, Heat map showing the motif enrichment in CRE clusters from **f**, as well as non-significant CREs (gray). **h**, Heat maps displaying median MPRA signals of matched CREs from posCor EGPs that contain a TF binding motif (wild type (WT)) or a scrambled version of it (Mut). Statistical significance was calculated using a paired Wilcoxon rank-sum test. *P < 0.01, **P < 0.001, ***P < 0.0001, ****P < 0.00001 **i**, Genomic tracks at the *Dll1* locus highlighting two positively correlated Neurog2-bound CREs (dashed box) that show the IPC-specific increase of chromatin accessibility (top) as well as MPRA signal (bottom bars). **j**, Detailed view of the two Neurog2-bound CREs indicated by dashed rectangles in **i**. NS, not significant.

**Extended Data Fig. 6:**
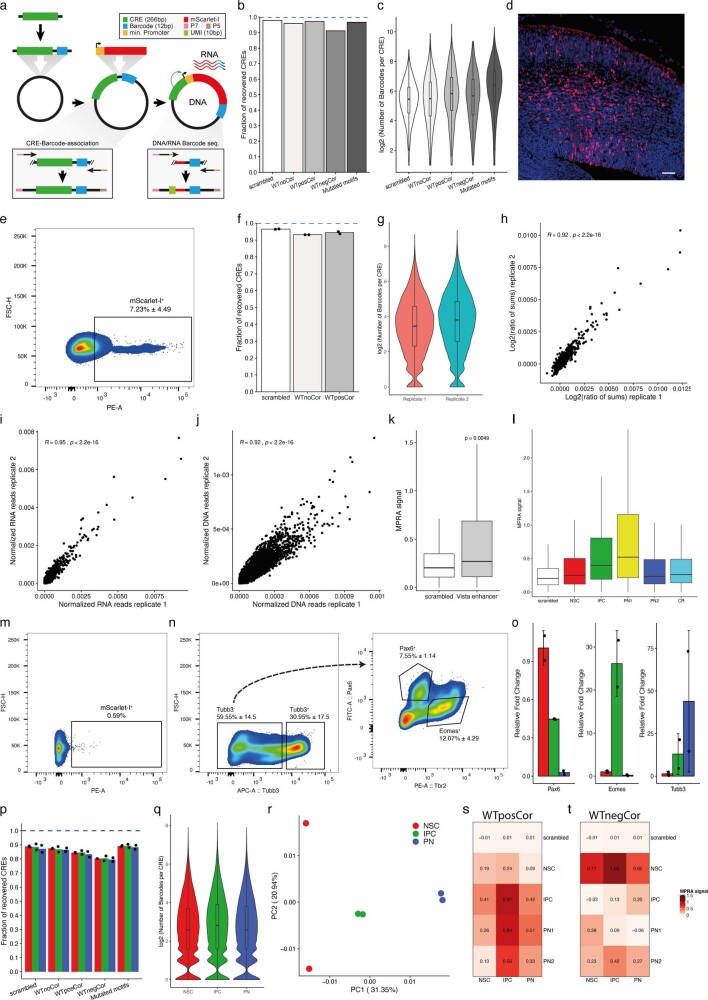
In vivo immunoMPRA validates cell type specific activity of identified CREs and their regulation by transcription factors. **(a)** Overview of MPRA pool generation and sequencing. **(b**) Barplot displaying the fraction of recovered CREs (≥1 unique barcode) in the MPRA plasmid pool. **(c)** Violin and box-whisker plots depicting the number of unique barcodes per CRE in the MPRA plasmid library. **(d)** Exemplary immunofluorescence image of a coronal section from a E15.5 neocortex 48h after IUE of the MPRA plasmid pool. red= mScarlet-I; blue= DAPI; Scale = 50μm **(e)** FACS strategy used to purify IUE electroporated mScarlet-I+ cells. A low stringency of the gate was chosen in order to capture also electroporated cells with lowly active MPRA plasmids. Number represent mean ± SD from the parental singlet population. **(f)** Barplot displaying the fraction of recovered CREs in DNA MPRA libraries. Dots represent biological replicates. **(g)** Violin and box-plots depicting the number of unique barcodes per CRE in DNA the MPRA libraries. **(h)** Scatter plot depicting log transformed ratio of sums for both biological replicates. **(i)** and **(j)** Scatter plot shows barcode read counts normalized to total read depth for RNA (i) or DNA (j) for each replicate. Numbers represent Pearson correlation coefficient and statistical significance (two-sided). **(k)** Box-plots depicts MPRA signal of scrambled control (white; n=1713) and CREs overlapping with VISTA forebrain enhancer (grey; n=81). Statistical significance is calculated using wilcoxon rank-sum test. **(l)** Box-plots depicts MPRA signal of scrambled controls (white; n=1713) and positive correlated enhancers separated by their cluster specificity (n for NSC=3439; IPC=777; PN1=1067; PN2=1566; CR=923). **(m)** Example FACS gate displaying the low residual mScarlet-I fluorescence (≤0.59%) after fixation. **(n)** Gating strategy for the immunoFACS of NSC, IPC and PN. Numbers represent mean ± SD from the parental singlet population. **(o)** Expression levels of NSC-(Pax6), IPC-(Eomes) and PN-(Tubb3) specific marker genes in the three isolated cell populations determined by RT-qPCR. Data are represented as a barplot showing the mean ± SD, as well as individual biological replicates (black dots; n=2). red= NSC, green= IPC, blue= PN. **(p)** Barplot displaying the fraction of recovered CREs (DNA) from NSC (red), IPC (green) and PN (blue). **(q)** Violin and box-whisker plots depicting the number of unique barcodes per CRE in both DNA and RNA MPRA libraries from isolated cell populations. **(r)** Principal component analysis performed on the ratio of sums for the replicates of NPC (red), IPC (green) and PN (blue). **(s-t)** Heatmaps displaying the mean MPRA signal per cell type for positive (s) and negatively (t) correlated CREs. In contrast to Fig. 4e cell type specificity of the enhancer-gene pairs was determined by maximal expression levels of the linked genes (see Methods). Box-plots (g,k,l) display median (line), 25^th^ or 75^th^ percentiles (box) as well as 10^th^ or 90^th^ percentiles (whisker).

To further characterize if the identified enhancers have the potential to drive gene expression, we performed an MPRA in vivo (Methods and Fig. 4a). We included 18,000 266-bp sequences (1,713 scrambled controls) and recovered ~95% of them during barcode association with an average of ~88 unique barcodes per enhancer (Extended Data Fig. 6a–c).

We then used in utero electroporation (IUE) to introduce the MPRA pool into the E13.5 embryonic cortex and sorted the electroporated cells 2 d after using fluorescence-activated cell sorting (FACS; Fig. 4a). This approach resulted in high reproducibility and barcode recovery (Extended Data Fig. 6d–j). Using MPRAnalyse^30^, we found that positively correlated enhancers were significantly more active compared to non-correlated enhancers and scrambled controls (Fig. 4b). Reassuringly, the sequences that partially overlap with VISTA forebrain enhancers^31^ were more active compared to scrambled controls, validating our approach (Extended Data Fig. 6k). Surprisingly, highly active enhancers were predominantly associated with neuronal genes, while NSC enhancers displayed low activity scores (Fig. 4c and Extended Data Fig. 6l), an observation we attributed to overrepresentation of differentiating neurons in the electroporated cells (Extended Data Fig. 6d,n).

To overcome this limitation and to obtain a cell-type-specific enhancer activity in vivo, we combined our IUE-based MPRA with an immunoFACS approach to isolate NSCs, IPCs and PNs (Methods and Extended Data Fig. 6m,n) and confirmed cell specificity by quantitative PCR (qPCR; Extended Data Fig. 6o). For all cell types, we achieved a high barcode recovery and a clear separation (Extended Data Fig. 6p–r). MPRA activity was highly correlated with enhancer cluster annotation for positively correlated (Fig. 4e and Extended Data Fig. 6s) but not negatively correlated pairs (Extended Data Fig. 6t).

To identify TFs that govern cell-type-specific reporter activity, we clustered all significantly active enhancers and performed motif enrichment analysis (Fig. 4f,g). We found enrichment of Tead binding motifs in the NSC-IPC cluster, neurogenic bHLH in IPC-PN cluster and AP-1 in NSCs/PNs, highly consistent with the results in Fig. 3c and the TFs expression patterns (Extended Data Fig. 5a–c). Conversely, inactive enhancers displayed a strong enrichment for NR4A2::RXRA and Hes TFs, which can act as transcriptional repressors^32,33^.

Next, we asked if TF binding directly regulates enhancer activity. For the majority of the analyzed TFs, mutating the motif was sufficient to strongly reduce reporter expression (Fig. 4h). Sequences containing the Neurog2 binding motif were associated with the highest overall signal and its mutation led to almost complete absence of reporter activity (Fig. 4h). This is exemplified at the *Dll1* locus, where the activity of IPC-specific *Dll1* enhancers was abolished upon mutation of the Neurog2 motif (Fig. 4i,j).

In summary, our in vivo MPRA assay not only expands existing databases of validated enhancer elements, but also allows us to make several important conclusions. First, the activity of the distal sequences identified in our positively correlated EGPs is largely cell-type specific and is significantly higher than comparable non-correlated sequences, validating our linkage method. Second, several TF motifs are associated with cell-type-specific reporter activity, which largely confirms and further extends our accessibility-based enrichment analysis. Finally, mutation of TF binding motifs abolishes reporter activity in a cell-type-specific manner, directly linking TF binding and enhancer function in vivo.

### Rewiring 3D genome organization and DNA methylation in vivo

**Fig. 5:**
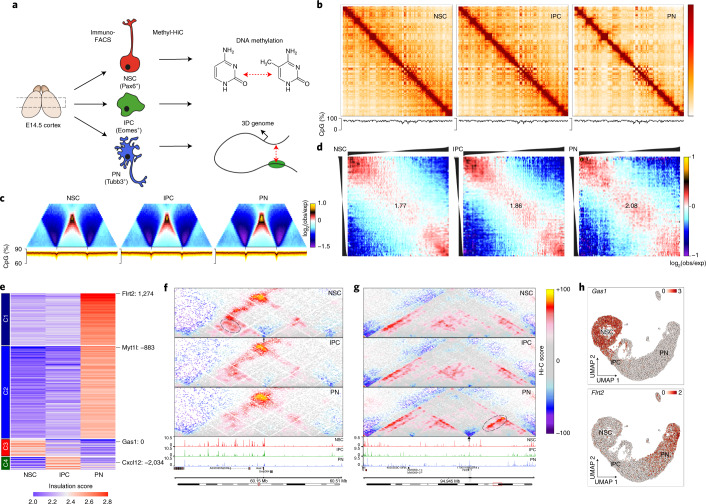
ImmunoMethyl-HiC identifies DNA methylation-independent global changes in 3D genome architecture during cortical development. **a**, Schematic representation of the immunoFACS Methyl-HiC experiment. **b**, Knight–Ruiz balanced contact matrices for chr3 at 250-kb resolution (top) and DNA methylation levels (bottom). **c**, Average contact enrichment (top) and DNA methylation levels (bottom) across TADs. **d**, Average contact enrichment between pairs of 250-kb loci arranged by their eigenvalue (shown on top). Numbers represent the compartment strength (Methods) **e**, *K*-means clustering of differential TAD boundaries (*n* = 322) based on insulation score. **f**,**g**, Contact maps (top) and aggregated accessibility of matched scATAC–seq clusters (bottom) for representative examples of an NSC-specific (**f**) or a PN-specific (**g**) TAD boundary (indicated by arrows) at the *Gas1* or *Flrt2* gene loci, respectively. Dynamic contacts are highlighted with dashed ellipses. **h**, scRNA UMAP projection, colored by the expression levels of indicated genes.

**Extended Data Fig. 7:**
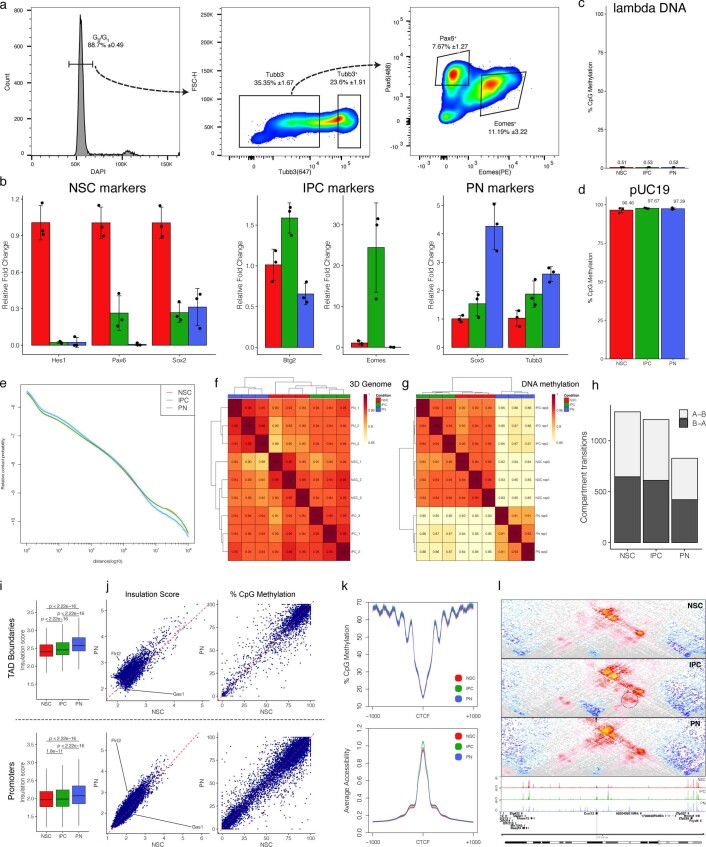
Global reorganization of the 3D genome in vivo during differentiation. **(a)** Gating strategy for the immunoFACS of NSC, IP and PN. Singlets in G_0_/G_1_ based on their DNA content (DAPI) were selected first (left), followed by separation of nuclei with high or low Tubb3 signal (middle). Tubb3 low cells were further subdivided based on Pax6 and Eomes (right). Number represent mean ± SD from the parental singlet population. **(b)** Expression levels of NSC- (Hes1, Pax6, Sox2), IPC- (Btg2, Eomes) and PN- (Sox5, Tubb3) specific marker genes in the three isolated cell populations determined by RT-qPCR. Data are represented as a bar plot showing the mean ± SD, as well as individual biological replicates (black dots). Red= NSC, green= IPC, blue= PN. n=3 biological replicates **(c-d)** Bar plots depicting methylation levels (mean ± SD) of the spiked-in bisulfite conversion controls: either completely unmethylated lambda DNA (c), or fully methylated pUC19 DNA. n=3 biological replicates. (d). Black dots indicate individual biological replicates. **(e)** Contact probability in logarithmic bins. Lines: mean values from biological replicates; semi-transparent ribbons: SEM. **(f-g)** Per sample pairwise Pearson’s correlation of either HiC contacts (f; at 50 kb resolution and considering only contacts separated by at least 100 kb) or DNA methylation levels (g; 500bp bins of union peakset). Note that the major separation occurs between cell types. **(h)** Stacked bar plot depicts cell type specific compartment transitions. **(i)** Box-plots indicate cell type specific insulation scores at TAD boundaries (top, n=4609) or gene promoters (bottom, n=23702). Plot displays median (line), 25^th^ or 75^th^ percentiles (box) as well as 10^th^ or 90^th^ percentiles (whisker). Statistical significance is calculated using a two-sided wilcoxon rank-sum test. **(j)** Scatterplot of insulation scores (left) and average DNA methylation levels (right) at TAD boundaries (top) and gene promoters (bottom) in the indicated cell types. **(k)** Average DNA methylation levels of the three isolated cell types (top) and average accessibility from matched scATAC-seq clusters (bottom) at CTCF peaks (NPC CTCF track). Note the nucleosomal pattern of DNA methylation around the CTCF sites. Lines: mean values from biological replicates; semi-transparent ribbons: SEM. **(i)** Contact maps (top) and aggregated accessibility of matched scATAC-seq clusters (bottom) for a representative example of an IPC specific TAD boundary (arrow) at the Cxcl12 gene locus. Dynamic contacts are highlighted with a dashed ellipse.

To obtain a more comprehensive view of how the chromatin landscape is reorganized during neural differentiation, we combined a modified Methyl-HiC^7,8^ approach (Methods and Supplementary Data 1) with immunoFACS of NSCs, IPCs and PN G_0_/G_1_ cells from E14.5 somatosensory cortex in biological triplicates (Fig. 5a and Extended Data Fig. 7a,b). Our improved Methyl-HiC method was characterized by high bisulfite conversion efficiencies (99.48%; Extended Data Fig. 7c,d), distance-dependent decrease in contact probability (Extended Data Fig. 7e), as well as a high reproducibility on the level of both the 3D genome (*r* ≥ 0.9; Extended Data Fig. 7f) and DNA methylation (*r* ≥ 0.93; Extended Data Fig. 7g). Furthermore, CTCF-associated methylation followed the expected pattern^34^ and did not change across cell types (Extended Data Fig. 7k).

Consistent with our previous results using an in vitro differentiation system^15^, we observed a global reorganization of chromatin interactions associated with fewer compartment transitions (Extended Data Fig. 7h) but stronger overall compartment strength (Fig. 5b,d), as well as increased insulation at topologically associating domain (TAD) boundaries and promoters upon neuronal differentiation (Fig. 5c and Extended Data Fig. 7i). This increased insulation was evident already in IPCs and was not associated with changes in DNA methylation at TAD boundaries or accessibility and DNA methylation at CTCF-bound sites (Fig. 5c and Extended Data Fig. 7i–k). We identified 322 differentially insulated domain boundaries (~11% of all; Fig. 5e and Supplementary Data 8), many of which were in close proximity to the transcription start site (TSS) of dynamically expressed genes such as *Gas1* (NSC), *Cxcd12* (IPC) or *Flrt2* (PN) and frequently associated with dynamic enhancer accessibility (Fig. 5f–h and Extended Data Fig. 7j).

**Fig. 6:**
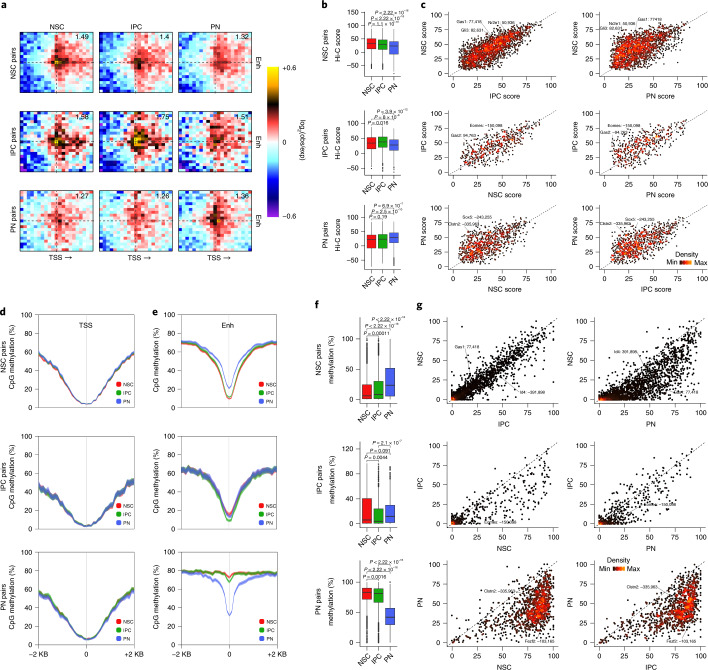
Dynamic enhancer–promoter loops and DNA methylation levels at regulatory regions. **a**, Aggregated Hi-C maps between enhancer (Enh) and TSS of intraTAD cluster-specific positively correlated EGPs. Genes are oriented according to transcription (arrow). Number in the top-right corner indicates the ratio of the center enrichment to the mean of the four corners (Methods). **b**,**c**, Box plots (**b**) and scatterplots (**c**), colored by density, depicting the interaction strength of intraTAD cluster-specific positively correlated E–P pairs. Statistical significance was calculated using a two-sided Wilcoxon rank-sum test. *n* = 4,116 NSC, 797 IPC and 1,571 PN EGPs. **d**,**e**, Average DNA methylation levels at TSSs (**d**) and enhancers (**e**) of cluster-specific positively correlated EGPs. Lines represent mean values from biological replicates; semitransparent ribbons denote the s.e.m. **f**,**g**, Box plots (**f**) and scatterplots (**g**) colored by density, depicting the average DNA methylation levels at enhancers of positively correlated cluster-specific pairs. Statistical significance was calculated using a two-sided Wilcoxon rank-sum test. *n* = 3,049 NSC, 672 IPC and 1,409 PN enhancers. All box plots display the median (line), 25th and 75th percentiles (box limits), 10th and 90th percentiles (whiskers) as well as outliers (dots).

**Extended Data Fig. 8:**
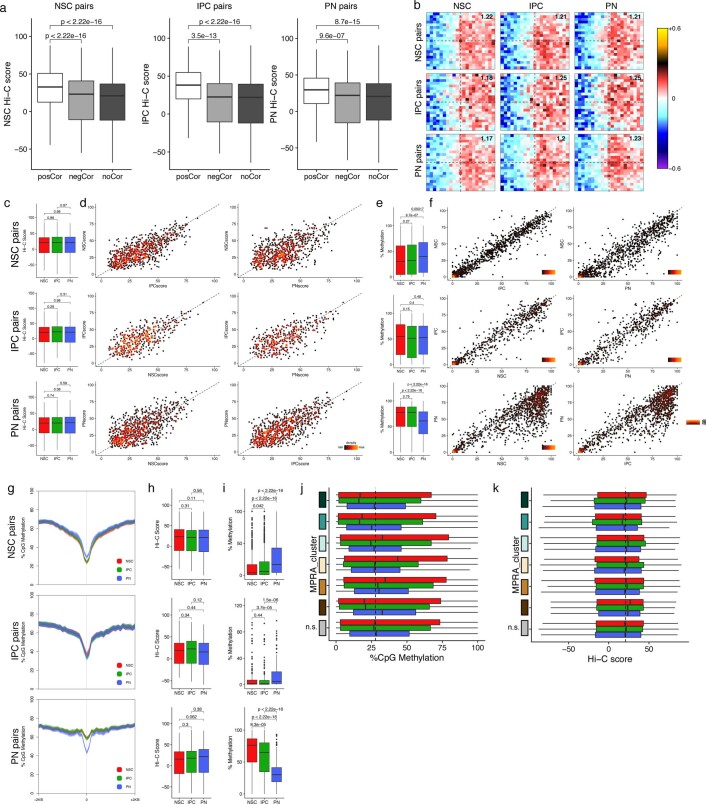
Non-correlated enhancer-gene pairs are not associated with dynamic chromatin looping or changes at DNA methylation levels. **(a)** HiC scores measured in NSC (left), IPC (middle) or PN (right) between cluster-specific positive (posCor), negatively (negCor) or non-correlated (noCor) enhancer-gene pairs indicating the strongest chromatin looping between posCor pairs. n= 4116 posCor, 1097 negCor and 1408 noCor NSC pairs; 797 posCor, 216 negCor and 797 noCor IPC pairs and 1571 posCor, 348 negCor and 1526 noCor PN pairs. Statistical significance is calculated using a two-sided wilcoxon rank-sum test. **(b)** Aggregated Hi-C maps between the linked distal peak and the transcription start site (TSS) of cluster specific randomly selected non-correlated (Control) enhancer-gene pairs (n=16978; Methods), displaying constant, cell type independent, low interactions between both. Genes are oriented according to transcription. Number in the top-right corner indicates the ratio of the center enrichment to the mean of the four corners (Methods). **(c-d)** Box- (c) and scatter plots colored by density (d), depicting the Hi-C score at non-correlated (Control) intraTAD enhancer-gene pairs at the indicated non-correlated cluster-specific pairs (Control). Statistical significance is calculated using a two-sided wilcoxon rank-sum test. n=1408 NSC, 797 IPC and 1526 PN EGPs. **(e-f)** Box- (e) and scatter plots colored by density (f), depicting the average DNA methylation levels at enhancers of non-correlated (Control) cluster-specific pairs. Statistical significance is calculated using a two-sided wilcoxon rank-sum test. n=1378 NSC, 782 IPC and 1487 PN noCor intraTAD enhancers. **(g)** Average DNA methylation levels at the enhancers (± 2kb) of non-correlated (Control) correlated cluster-specific pairs. Lines: mean values from biological replicates; semi-transparent ribbons: SEM. **(h-i)** Box-plots depicting the Hi-C scores at intraTAD peak-gene pairs (h, n=1097 NSC, 216 IPC and 348 PN EGPs) or DNA methylation levels (I, n=897 NSC, 183 IPC and 290 PN regions) at enhancers of intraTAD negative correlated cluster-specific pairs. Statistical significance is calculated using a two-sided wilcoxon rank-sum test. **(j)** Box-plots display cell-type-specific DNA methylation levels at positive correlated enhancers clustered according to their enhancer activity in the in vivo immunoMPRA assay (see Fig. 4f) **(k)** Analogous to (j) but displaying the cell-type-specific HiC-scores between enhancer and associated gene. Box-plots (a, c, e, h-k) display median (line), 25^th^ or 75^th^ percentiles (box), 10^th^ or 90^th^ percentiles (whisker) as well as outliers (dots; only i).

To address if there is a global rewiring of regulatory interactions, we examined the aggregated Hi-C E–P contacts for each cluster (Supplementary Data 6) based on the previous EGP definitions (Fig. 3a). We found that positively correlated E–P pairs were characterized by a higher overlap with chromatin loops (42.78% versus 27.75%; *P* ≤ 0.00001, Fisher’s exact test) and increased contact strength compared to negatively correlated and non-correlated E–P pairs (Extended Data Fig. 8a). Furthermore, positively correlated E–P loops were largely cell-type specific, with the highest contact strength in the cell type where the enhancer is the most active (Fig. 6a,b).

Next, we confirmed that only changes in positively correlated, but not in control pairs were statistically significant (Fig. 6b and Extended Data Fig. 8b,c). However, individual E–P pairs were characterized by a continuum of contact strengths including pre-looping or even anti-correlated with changes in accessibility and expression (Fig. 6c). Examples of dynamic E–P contacts include cell-type-specific genes such as *Gas1* (also depicted in Fig. 5f), *Gli3* and *Nr2e1* in NSCs, Eomes and *Gas2* in IPCs and neuronal genes such as *Sox5* and *Clstn2*.

To understand how DNA methylation is related to changes in enhancer accessibility and gene expression, we analyzed the dynamics of CpG methylation at enhancers and promoters. We found that promoter regions displayed consistent hypomethylation levels throughout neurogenesis (Fig. 6d), while DNA methylation levels were lowest at accessible enhancers (Fig. 6e,f), consistent with previous reports^35,36^. While the majority of enhancers became consistently hypomethylated upon activation, DNA methylation levels varied considerably (Fig. 6g and Extended Data Fig. 8e–g). Although chromatin interactions between negatively correlated pairs did not change significantly, DNA methylation levels were variable, suggesting that these two molecular layers are not strictly correlated (Extended Data Fig. 8h,i). Furthermore, MPRA-based enhancer activity and DNA methylation levels (or interaction strength) were also only weakly correlated, consistent with the lack of locus-specific context in MPRA assays (Extended Data Fig. 8j,k).

In summary, we show that most regulatory interactions between EGPs are fairly dynamic and strongest in the cell type where the enhancer is active and the linked gene expressed. Despite this overall trend, considerable heterogeneity exists at the level of individual E–P pairs; while many follow the classical model of cell-type-specific looping, some appear to be already pre-looped before transcription occurs and others are only weakly interacting with their target promoters. DNA methylation levels at enhancers are generally anti-correlated with accessibility but, again, vary considerably from enhancer to enhancer. Importantly, neither 3D contact strength nor DNA methylation could be inferred simply from scRNA-seq, scATAC–seq or MPRA data, thus highlighting the importance of integrating multiple epigenome layers to study GRNs.

### Transcription factor-associated 3D epigenome remodeling and DNA demethylation

Given the observed dynamics and heterogeneity at the identified EGPs, we sought to identify the underlying molecular mechanisms. TFs have been previously suggested as potential mediators of chromatin looping by us and others^9,15,37,38^, but it is unclear which ones are important and to what extend they participate in coordinated epigenome remodeling.

**Fig. 7:**
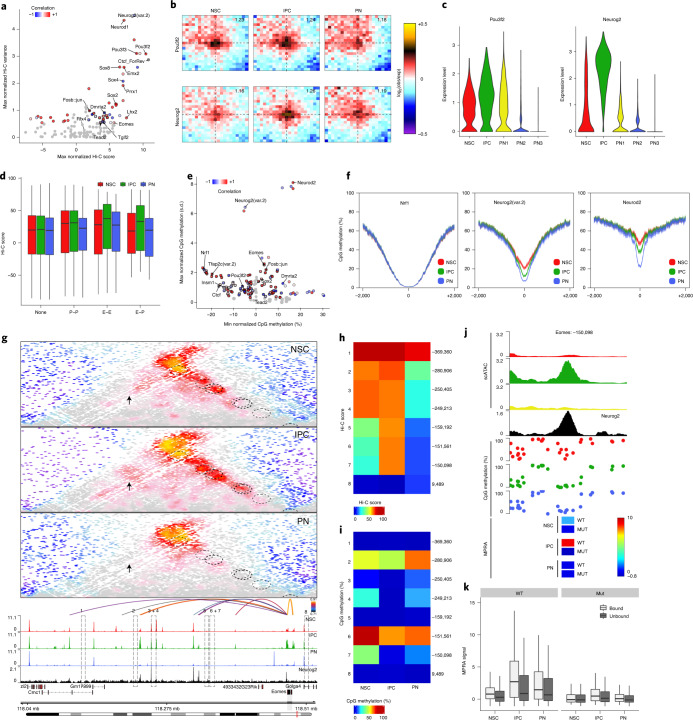
Transcription factors are associated with changes in both chromatin looping and DNA methylation. **a**, Scatterplots depicting the maximum Hi-C score and the normalized variance at pairs of accessible peaks associated with specific TF binding motifs. TFs are colored based on the Pearson correlation between their expression and the accessibility of their binding motif. Gray circles represent non-significant TFs (*P* > 0.05; permutation test). **b**, Aggregated Hi-C plots between intraTAD pairs of accessible peaks overlapping with the indicated TF motif. **c**, Violin plots depicting the expression levels of indicated TFs per scRNA cluster. **d**, Box plots depicting cell-type-specific Hi-C scores between pairs of Neurog2-bound promoters (P–P; *n* = 1,298), enhancers (E–E; *n* = 298) enhancer–promoters (E–P; *n* = 49) or none (*n* = 13,114). **e**, As in **a** but comparing the minimum DNA methylation level and the normalized standard deviation at accessible peaks associated with specific TF binding motifs. **f**, Average DNA methylation levels at accessible peaks overlapping with the indicated TF binding motifs. Lines denote mean values from biological replicates; semitransparent ribbons represent the s.e.m. **g**, Contact maps (top) and aggregated accessibility of matched scATAC–seq clusters (bottom) at the *Eomes* locus. Depicted are the identified linked enhancers (1–8; arcs/circles) and Neurog2 ChIP–seq track. Arcs on top are colored by the Pearson correlation of the enhancer accessibility and Eomes expression. Arrows point to E–E interactions. **h**,**i**, Heat maps depicting the Hi-C score of linked enhancer–Eomes pairs (**h**) or the DNA methylation levels at the corresponding Eomes enhancers (**i**). **j**, Detailed view of the Eomes: −150,098 enhancer depicting accessibility, Neurog2 ChIP–seq (top) and DNA methylation levels (middle) as well as cell-type-specific MPRA signal of the WT or mutated (MUT) sequence. **k**, Box plots displaying the cell-type-specific MPRA signal of Neurog2-bound (light gray; *n* = 154 for each NSC, IPC and PN category) or unbound (dark gray; *n* = 784 for each NSC, IPC and PN category) enhancers with either WT or mutated motif sequence (Mut; right). The box plots (**d** and **k**) display the median (line), 25th and 75th percentiles (box limits) as well as the 10th and 90th percentiles (whiskers).

**Extended Data Fig. 9:**
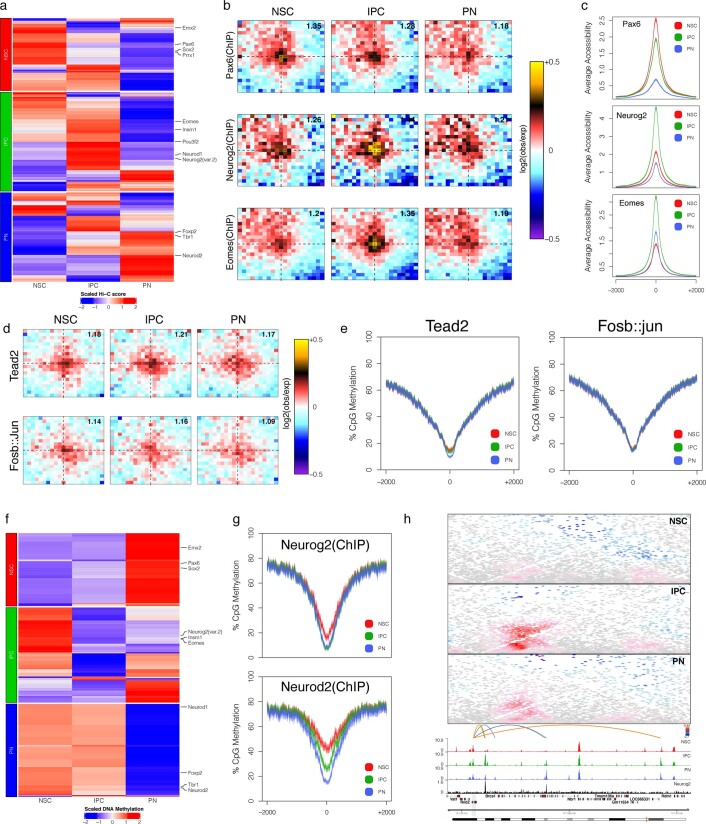
Transcription factors associated with cell-type-specific looping based on positively correlated enhancer-gene pairs. **(a)** Heatmaps depicting the scaled Hi-C score at positively correlated cluster-specific enhancer-gene pairs, where the TF motifs overlap with both the enhancer and the promoter. Only significant (p<0.05, permutation test) and expressed TFs are displayed. **(b)** Aggregated Hi-C plots of intraTAD pairs of ChIP-seq based bound sites. Number in the top-right corner indicates the ratio of the center enrichment to the mean of the four corners (Methods). **(c)** Average accessibility per cluster centered at the indicated ChIP-seq based binding sites. **(d)** As (b) but for motif based bound sites of indicated transcription factors. **(e)** Average DNA methylation levels centered at motif based Tead2 and Fosb::Jun binding sites. **(f)** As (a) but plotting the scaled DNA methylation levels at enhancers, overlapping with TF motifs. **(g)** As (e) but with centered ChIP-seq based Neurog2 and Neurod2 binding sites. **(h)** Contact maps (top) and aggregated accessibility of matched scATAC-seq clusters (bottom) at the Rnd2 locus. Arcs represent positively correlated enhancers-Rnd2 pairs and are colored based on the correlation between the enhancer accessibility and Rnd2 expression. Shown is also Neurog2 ChIP-seq track. Note the IPC-specific chromatin loops established between Neurog2-bound enhancers and Rnd2 promoter.

To address this question, we determined the average interaction strength and specificity for each TF based on accessible sites containing their binding motif (Methods). Surprisingly, we found several TFs associated with strong looping, cell-type specificity or both (Fig. 7a, Extended Data Fig. 9a and Supplementary Data 9). Some (such as members of the POU domain (class 3) and Sox families) have been previously described in the context of human brain development^9^, while others (such as Neurog2 and Dmrta2) have not yet been implicated in the context of the 3D genome. To confirm these findings, we plotted the aggregated Hi-C contact maps for Pou3f2 and Neurog2 motifs (Fig. 7b) or ChIP–seq-based peaks (Extended Data Fig. 9b) and observed that interaction strength was highly correlated with TF expression (Fig. 7c). This dynamic pattern of chromatin interactions matched well with the changes in accessibility at TF bound sites (Extended Data Fig. 9c) and it was in contrast with the relatively weaker and static loops associated with other TFs such as AP-1 and Tead (Extended Data Fig. 9d,e). Finally, we observed dynamic looping not only between Neurog2-bound enhancers and promoters, but also between pairs of Neurog2-bound enhancers (Fig. 7d).

Next, we used an analogous approach to identify TFs related to dynamic DNA methylation. We identified Neurod2 (previously linked to DNA demethylation in the cortex^35^) and Neurog2 among others (Supplementary Data 9) to be associated with high cell-type variability (Fig. 7e,f) and confirmed these results using publicly available ChIP–seq data (Extended Data Fig. 9f,g).

Finally, we focused on Neurog2, as it was one of the few TFs that was correlated with dynamic chromatin interactions as well as changes in DNA methylation and due to its well-characterized role in neuronal differentiation in the cortex^39^. This can be exemplified at the *Eomes* locus where Neurog2-bound enhancers engaged in strong, cell-type-specific looping with each other and with the *Eomes* promoter only in IPCs (Fig. 7g,h; loci 5–7), while other enhancers were either pre-looped (Fig. 7g,h; loci 1–4) or weakly interacting (Fig. 7g,h; locus 8). DNA methylation levels at Neurog2-bound enhancers were either very low (Fig. 7i, locus 5) or decreased specifically in IPCs (Fig. 7i; loci 4, 6 and 7). Focusing on enhancer 7 as an example (Eomes: −150,098), we could validate its cell-type-specific MPRA reporter activity in IPCs, which was completely abolished upon mutating the Neurog2 motif (Fig. 7j), indicating direct regulation consistent with previous findings^40,41^. The relationship between enhancer activity and Neurog2 binding was also confirmed genome wide across all tested Neurog2-bound enhancers (Fig. 7k).

Collectively, these experiments identify the previously underappreciated role of TFs in dynamic chromatin looping and DNA methylation. Several TFs with a well-characterized role in cortical development such as Neurog2, Pou3f2 and Eomes are associated with strong cell-type-specific looping, but only Neurog2 binding is also correlated with changes in DNA methylation. We further demonstrate that the activity of Neurog2 enhancers directly depends on its binding genome wide.

### Neurog2 is sufficient to induce epigenome changes in vivo

**Fig. 8:**
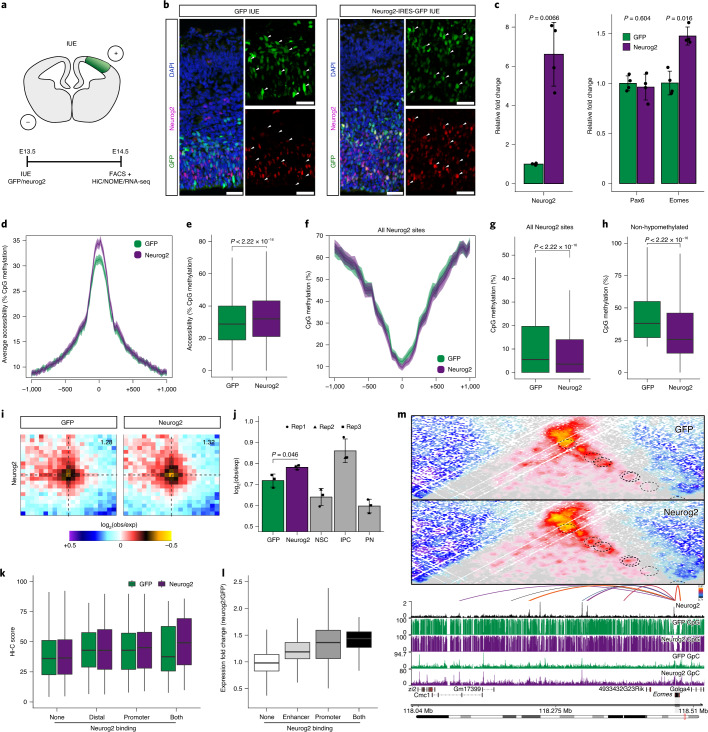
Neurog2 is sufficient to induce multilayered epigenome changes in vivo. **a**, Schematic representation of the experimental design. **b**, Representative immunofluorescence images for GFP and Neurog2 24 h after control (GFP only) and overexpression (Neurog2-IRES-GFP) electroporations. Scale bars, 50 µm. **c**, Expression levels were determined by qPCR with reverse transcription. Data are represented as a bar plot showing the mean ± s.d., as well as individual biological replicates (black dots; *n* = 4). Statistical significance was determined using a two-sided paired *t*-test. **d**, Average accessibility at Neurog2 ChIP–seq peaks. Lines represent mean values from biological replicates; semitransparent ribbons denote the s.e.m. **e**, Box plots showing the average accessibility within a 200-bp window around Neurog2 peak centers (*n* = 2,172 regions). Statistical significance was calculated using a two-sided Wilcoxon paired rank-sum test. **f**, As in **d** but plotting DNA methylation values. **g**, As in **e** but plotting DNA methylation values (*n* = 1,168). **h**, Box plots showing the average accessibility within a 200-bp window around non-hypomethylated Neurog2 peak centers (%CpG ≥ 20 in the control condition; *n* = 296 regions). Statistical significance was calculated using a two-sided paired Wilcoxon rank-sum test. **i**, Aggregated Hi-C plots between intraTAD pairs of Neurog2 peaks. **j**, Quantification of the contact enrichment at intraTAD pairs of Neurog2 peaks (100 kb–2 Mb apart; *n* = 3). Statistical significance was calculated using a two-sided paired *t*-test. **k**, Box plots displaying the Hi-C score between positively correlated E–P pairs bound by Neurog2 at promoter (*n* = 348), enhancer (*n* = 655), none (*n* = 15,234) or both (*n* = 56). **l**, Analogous to **k** but displaying the expression fold change between control and Neurog2 overexpression. **m**, Contact maps (top) and methylation tracks (bottom) at the *Eomes* locus. Depicted are the identified linked enhancers (arcs) and Neurog2 ChIP–seq track. All box plots (**e**, **g**, **b**, **k** and **l**) display the median (line), the 25th and 75th percentiles (box limits), as well as the 10th and 90th percentiles (whiskers).

**Extended Data Fig. 10:**
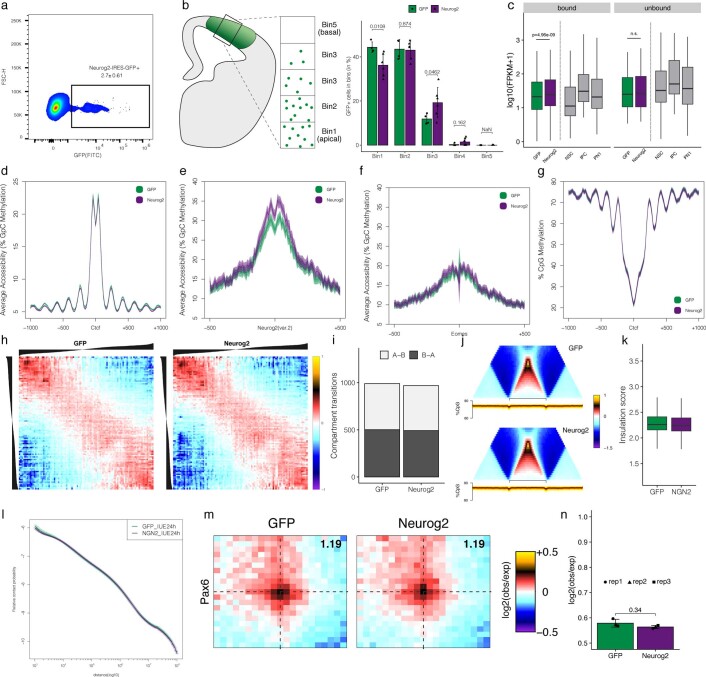
No global reorganization of chromatin accessibility, DNA methylation and 3D genome architecture upon Neurog2 overexpression. **(a)** FACS strategy used for purifying Neurog2-IRES-GFP electroporated cells after 24h. Number represent mean ± SD from the parental singlet population. **(b)** Schematic (left) and quantification (right) of the distribution of GFP^+^ electroporated cells across 5 equal size bins, spanning the whole cortical area. Black dots indicate individual replicates. Statistical significance is calculated using unpaired t-test. **(c)** Boxplots depict expression levels of Neurog2 bound (n=133) or unbound (n=33) IPC-specific genes based on its motif in the indicated samples or aggregated scRNA-seq clusters (grey boxplots). Statistical significance is calculated using a two-sided wilcoxon rank-sum test. **(d-f)** Average accessibility at Ctcf peaks Neurog2 motifs overlapping with Neurog2 peaks, or Eomes motifs overlapping with Eomes peaks. Lines: mean values from biological replicates; semi-transparent ribbons: SEM. **(g)** As (d) but for average DNA methylation levels. **(h)** Average contact enrichment between pairs of 250 kb loci arranged by their eigenvalue (shown on top). **(i)** Stacked barplots depicting compartment transitions. **(j)** Average contact enrichment and DNA methylation levels at TADs. **(k)** Boxplot showing the insulation scores at TAD boundaries of the indicated samples. n=4609 **(l)** Contact probability in logarithmic bins. Lines: mean values from biological replicates; semi-transparent ribbons: SEM. **(m-n)** Aggregated Hi-C plots and bar-plot displaying quantification of intraTAD pairs of Pax6-bound sites. Statistical significance is calculated using a two-sided paired t-test. n=3. Box-plots (c, k) display median (line), 25^th^ or 75^th^ percentiles (box) as well as 10^th^ or 90^th^ percentiles (whisker).

To understand the mechanism that links Neurog2 binding to changes in DNA methylation, chromatin accessibility and 3D regulatory loops, we used a gain-of-function approach in vivo (Fig. 8a,b and Extended Data Fig. 10a).

First, we verified Neurog2 overexpression by qPCR and confirmed that *Pax6* levels were not affected (Fig. 8c), while the expression of the direct target *Eomes*^40^ was increased. Consistent with previous findings^29^, Neurog2 overexpression was also associated with increased neuronal migration (Extended Data Fig. 10b). RNA-seq confirmed that among IPC-specific genes, only those bound by Neurog2 were upregulated, suggesting negligible fate shift from NSC to IPC within the 24-h time window of the experiment (Extended Data Fig. 10c).

Next, we asked if Neurog2 overexpression affects the local chromatin accessibility and DNA methylation. Neurog2 binding sites and Neurog2 motifs became more accessible upon Neurog2 overexpression (Fig. 8d,e and Extended Data Fig. 10e), while Ctcf sites or Eomes motifs were not affected (Extended Data Fig. 10d,f). These results are consistent with our computational predictions (Fig. 3c) and the proposed pioneering activity of Neurog2 (ref. ^42^). In addition to changes in accessibility, Neurog2 overexpression also led to DNA demethylation at its bound regions (Fig. 8f–h) but not at Ctcf sites (Extended Data Fig. 10g).

Next, we addressed if Neurog2 binding is sufficient to affect chromatin looping. We observed that Neurog2-bound sites interacted stronger upon Neurog2 overexpression (Fig. 8i,j), while there was no change at Pax6 binding sites (Extended Data Fig. 10m,n). Furthermore, global 3D genome topology at the level of TADs, compartments and contact probability was not affected (Extended Data Fig. 10h–l), suggesting that Neurog2 overexpression does not lead to IPC-like nuclear architecture. Importantly, only positively correlated E–P pairs bound by Neurog2 at both anchors were characterized by increased interaction strength (Fig. 8k). Taken together, these results suggest that Neurog2 binding is sufficient to induce chromatin looping between regulatory regions within 24 h.

Finally, we examined the consequences of Neurog2-mediated regulatory contacts for gene expression. Consistent with the earlier findings, we observed that expression at unbound genes was generally unchanged upon Neurog2 overexpression, arguing against secondary effects (Fig. 8l). Genes where Neurog2 binding occurred only at the paired enhancer were only slightly upregulated, while Neurog2 present at the promoter led to a relatively stronger increase in expression (Fig. 8l). We observed the highest upregulation for genes where Neurog2 was bound both at the paired enhancer and promoter (Fig. 8l), indicating a potential synergistic effect.

One example of such locus is *Eomes*, where we observed specific increase of E–P contacts upon Neurog2 overexpression only at bound enhancers (Fig. 8m). DNA methylation and chromatin accessibility were affected at some, but not all, Neurog2-bound Eomes enhancers, suggesting that the increase in contacts is not a direct consequence of changes in the linear epigenome.

Collectively, these experiments confirm our computational predictions and identify a previously unknown role of Neurog2 in regulating dynamic chromatin looping and DNA methylation. Importantly, Neurog2 can mediate regulatory interactions only when it is bound at both anchors, that is, the enhancer and the promoter. Finally, we show that genes where such a binding pattern occurs are more robustly upregulated upon Neurog2 overexpression.

## Discussion

Here, we combined single-cell transcriptomics and chromatin accessibility with cell-type-specific massively parallel reporter activity, DNA methylation and 3D genome architecture in vivo to address how changes across multiple regulatory layers can be coordinated to facilitate specific lineage decisions in the mouse cortex.

Integrating matched scRNA and scATAC data, we identified thousands of positively correlated EGPs, generating a rich resource to study GRNs. We showed that although enhancer activation generally precedes transcription at dynamic genes, only a subset of enhancers appears to be truly lineage priming. These findings corroborate prior conclusions in early mammalian embryogenesis^6^ and recent data using simultaneous paired ATAC/RNA measurements in single cells^43^. The presence of neural bHLH and T-box TFs at primed enhancers suggests a pioneering function; however, priming of Neurog2-bound regions was associated specifically with Meis1 motifs and the absence of Hey/Hes binding. These results suggest that chromatin context, enhancer grammar and cofactors may influence pioneering activity of Neurog2, similarly to how co-binding can shape the activity of other pioneering factors such as Foxa2 in the endoderm^44^.

Using a modified MPRA approach, capable of quantifying cell-type-specific enhancer activity in vivo, we could confirm our integration-based linkage method and expand the repertoire of validated neuronal enhancers. We identify several TFs which are associated with cell-type-specific reporter activity (such as AP-1 and Tead for NSCs and neural bHLH TF in IPCs and PNs) and confirm their direct effect using motif mutagenesis. Focusing on Neurog2, we show that higher reporter activity is associated with stronger Neurog2 binding, and mutation of Neurog2 motif abolishes this correlation.

Three-dimensional genome architecture and DNA methylation represent two epigenome layers that are important for gene regulation, but have not yet been coupled to single-cell transcriptomic and accessibility measurements^3,4^. Our immunoFACS approach allowed us to combine the resolution and depth of bulk Hi-C/DNA methylation with cell-type specificity. The reduced input requirements and the improved resolution compared to previous studies^7,8^ enable the application of this method to other tissues and model organisms.

Previous studies have produced somewhat conflicting results regarding the importance of 3D genome architecture on gene regulation^45–48^ and the cell-type specificity of regulatory loops^9,14,15,49,50^. Our results indicate that contact strength between enhancers and promoters (as well as DNA methylation levels) is not simply a consequence of gain in accessibility/expression, as even highly correlated EGPs were characterized by a continuum of interactions scores. Despite this apparent heterogeneity, the majority of E–P interactions appear to be dynamic and cell-type specific, yet such differences are rather quantitative than binary. Furthermore, the ability of a sequence to enhance expression in a reporter assay (the ‘classical’ definition of enhancer) cannot be directly predicted from other epigenome layers such as accessibility, looping or DNA methylation.

Finally, we provide evidence that a subset of TFs is associated with dynamic chromatin looping, potentially acting as ‘molecular bridges’ to facilitate and coordinate epigenome remodeling. We identify the proneural TF Neurog2 as one such factor and show that it is sufficient to induce local chromatin accessibility and DNA demethylation at its binding sites in vivo. The simultaneous binding of Neurog2 at both the enhancer and promoter, as well as the increased chromatin looping associated with such configuration, leads to stronger transcriptional upregulation of the downstream target genes. Thus, regulatory homotypic interactions mediated by TFs may ensure more robust changes in gene expression and cell fate in a dynamic developmental system. Such interactions can be achieved via multimerization^51^, transient phase transitions^52,53^ or via cofactors such as Lmo4/Ldb1 (refs. ^45,54^).

Although the cell types we focused on belong to temporally separated lineages (NSCs at E14.5 give rise to superficial layer neurons and glia cells, while PNs have primarily deep layer identity), the focus of our work is on the epigenome remodeling associated with conserved neuronal differentiation GRNs. Indeed, very recent findings on the molecular logic of subtype diversification in the cortex^3^ conclude that the neural differentiation programs are mostly conserved and divergence occurs primarily in postmitotic neurons.

Collectively, our data provide a comprehensive view of the reorganization of the epigenetic regulatory landscape during lineage commitment in the mouse cortex. Our results pave the way for functional studies aiming to resolve the relative influence of dynamic versus pre-looped enhancers on gene expression and the mechanism of how TFs such as Neurog2 can rewire the regulatory 3D genome. Our functional experiments also suggest that E–P homotypic interactions stabilized by TFs, as well as multimodal epigenome remodeling can lead to the robustness of the transcriptional response and lineage specification.

## Methods

### Plasmids

An IRES-eGFP cassette, with or without Neurog2, was introduced in the dCas9 control plasmid^36^ (kind gift from the Calegari laboratory) with prior removing of the U6-gRNA scaffold and the 3xFlag-dCas9-T2A-eGFP sequence. Neurog2 was amplified from cDNA of E14.5 mouse cortices, and all primers used for cloning are listed in the Supplementary Data 10.

### MPRA design and plasmid pool generation

A detailed protocol for the MPRA plasmid pool generation can be found at https://www.protocols.io/view/mpra-plasmid-pool-preparation-bxchpit6/.

The designed MPRA plasmid pool included 7,772 enhancers associated with positively correlated EGPs, 1,679 negatively correlated EGPs or 873 non-correlated EGPs, as well as 1,713 scrambled control sequences, which had matched GC content and were prescreened to minimize the presence of expressed TF motifs. Additionally, we added 4,398 posCor sequences where only the corresponding motif sequence was iteratively mutated (100 permutations with similar GC content, lowest motif score selected). All sequences were centered and resized to 266 bp and extracted using the ‘BSgenome.Mmusculus.UCSC.mm10’ R package. To facilitate barcode–CRE association we added a 4 bp tag at the beginning of each WT/control (ATTA) or Mut (TCCG) sequence.

In total, 266-bp single-stranded oligonucleotides were synthesized (Twist Bioscience) and degenerated barcodes as well as KpnI/EcoRI restriction sites were added by two separate PCRs. PCR products were introduced into the pMPRA1 (ref. ^55^; Addgene plasmid no. 49349) backbone via Gibson assembly and transformed into ElectroMAX Stbl4 Competent Cells (Thermo Fisher) using Gene Pulser/MicroPulser Electroporation Cuvettes with a 0.1-cm gap (parameters: 1.8 kV, 25 µF, 200 Ω). Transformed bacteria were immediately resuspended in 1 ml of warm SOC medium and a 1:10 dilution of the bacteria was distributed on ten plates of LB agar containing 100 µg µl^−1^ carbenicillin. The number of transformants was estimated on a 1:10.000 diluted counting plate, and 1.2 million colonies, corresponding to approximately 68 barcodes per CRE, were scraped for plasmid purification. The purified plasmids were digested with KpnI/EcoRI and ligated with an insert containing the minimal promoter (derived from pNL3.1 (Nluc/minP), Promega) and mScarlet-I (kind gift of the laboratory of M. Götz). The purified ligation product was transformed in *Escherichia coli* as described above, scraped and the final plasmid library was purified using the EndoFree Plasmid Maxi Kit (Qiagen).

### Experimental model and subject details

Time-mated pregnant C57BL/6JRj mice were obtained from Janvier Laboratories and kept under standard housing conditions according to local regulations of the Regierung Oberbayern, Germany. E14.5 mouse embryos were used independent of sex. Each biological replicate represents a single embryo from different mothers in case of scRNA-seq/scATAC–seq or a pool of 4–6 littermates from separate mothers in the case of Methyl-HiC. For IUE experiments, matched biological replicates consisted of 1–3 GFP and Neurog2 electroporated littermates from the same mother or 3–5 littermates for the MPRA pool. All experiments were performed according to national guidelines and were approved by local authorities (Regierung Oberbayern, Germany; ROB-55.2-2532.Vet_02-19-175). No animals or data points were excluded from the analysis.

### In utero electroporation

IUE was performed as previously described^36^. Briefly, E13.5 time-mated plugs were anesthetized using isoflurane, the uterus was exposed and 1–3 µl plasmid DNA in PBS was injected into the lumen of the telencephalon followed by five pulses of 35 V, 100 ms each, at 1-s intervals delivered through platinum electrodes (NepaGene). At 24 or 48 h after electroporation, the pregnant females were euthanized, the brains of the embryos dissected and the electroporation efficiency evaluated using a fluorescence stereotactic microscope.

### Tissue preparation and dissociation

Brains of WT or electroporated E14.5 and E15.5 embryos were either used directly for in situ hybridization and immunohistochemistry or further dissected to isolate the somatosensory cortex with a prior removal of all meninges. The dissected cortex was dissociated using a papain-based neural dissociation kit (Miltenyi Biotec) according to the manufacturer’s protocol. The cell number and viability were assessed using a Countess II Automated Cell Counter (Invitrogen).

### Sectioning, immunohistology and in situ hybridization

Brains were fixed in freshly prepared 4% formaldehyde in PBS at 4 °C for at least 8 h, washed once in PBS and cryoprotected in 30% sucrose for ~6 h at 4 °C. Subsequently, brains were embedded in TFM-Tissue Freezing Media (TBS; Triangle Biomedical), snap frozen on dry ice and finally cryosectioned (~10 μm) using a CryoStar NX70 (Thermo Fisher). Sections were collected on Superfrost Plus adhesive microscope slides (Thermo Fisher) and stored at −80 °C until further use.

For immunohistochemistry, the sections were hydrated in PBS and incubated for 1 h at room temperature (RT) in PBS blocking buffer containing 5% horse serum (Sigma-Aldrich), 1% BSA (Thermo Scientific) and 0.3% Triton X-100 (Sigma-Aldrich). Staining was performed overnight at 4 °C with anti-GFP (1:1,000 dilution; Abcam) and anti-Neurog2 (1:250 dilution; Cell Signalling) antibodies in PBS blocking buffer. Sections were washed three times for 10 min with 0.1% Triton X-100 in PBS and, if required, stained with secondary antibodies (1:1,000 dilution; goat anti-chicken-A488 and donkey anti-rabbit-A555, Thermo Scientific) followed by an incubation with DAPI (1:1,000 dilution in PBS) for 10 min and finally mounted using Fluoromount-G (Invitrogen).

RNA in situ hybridization was performed using the RNAscope Multiplex Fluorescent Reagent kit v2 (ADCBio) according to the instruction manual. Signal amplification and detection reagents, including Opal fluorophores (Akoya Biosciences), were applied sequentially. Nuclei were counterstained with DAPI and slides mounted with Fluoromount-G (Invitrogen).

All images were acquired using a Zeiss LSM 710 confocal microscope. Data collection and analysis were not performed blind to the conditions of the experiments.

### scRNA-seq and scATAC–seq

scRNA-seq (v3, 10x Genomics) as well as scATAC–seq (10x Genomics) libraries were generated according to the instruction manual with a targeted recovery of 6,000 cells per nuclei for each sample.

### Intracellular immunostaining

A detailed protocol for the immunoFACS can be found at https://www.protocols.io/view/immunofacs-b2a2qage/. Briefly, dissociated cells were fixed for 10 min at RT in 1% freshly prepared formaldehyde in PBS (Thermo Fisher) and quenched by addition of glycine (Invitrogen) to a final concentration of 0.2 M. Fixed cells were spun down with 500*g* for 5 min at 4 °C, washed once with 1% BSA, 0.1% RNasin plus RNase inhibitor (Promega) in PBS (wash buffer) and subsequently incubated for 10 min at 4 °C in permeabilization buffer consistent of 0.1% freshly prepared Saponin (Sigma-Aldrich), 0.2% BSA (Thermo Fisher) and 0.1% RNasin plus RNase inhibitor in PBS. Staining against Pax6 (1:40 dilution; BD Bioscience), Eomes (1:33 dilution; BD Bioscience) and Tubb3 (1:13 dilution; BD Bioscience) was performed in staining buffer (0.1% saponin, 1% BSA and 0.1% RNasin plus RNase inhibitor in PBS) for 1 h at 4 °C under slow rotation. Cells were washed twice with permeabilization buffer, once with wash buffer containing DAPI (1:1,000 dilution; Thermo Fisher, 62248) and a final wash with wash buffer without DAPI. Between each washing step, the cells were incubated for 5 min at 4 °C with the respective buffer under slow rotation and spun down with 2,500*g* for 5 min at 4 °C. After the last wash, the cells were resuspended in PBS with 1% BSA and 1% RNasin plus RNase inhibitor, passed through a 40-µm cell strainer (Thermo Fisher) and immediately FACS sorted.

### Fluorescence-activated cell sorting

FACS was performed on a BD FACSAria Fusion (BD Bioscience) with four lasers (405, 488, 561 and 640 nm) using a 100-µm nozzle. Singlets of immunostained cells were selected using forward and side scatter, cells in G_0_ and G_1_ were identified by genomic content based on DAPI staining. Subsequently, these cells were divided into Tubb3-high for PNs and low for progenitor cell types. The progenitor population was further subdivided into Pax6-high/Eomes-low for NSCs and Eomes-high for IPCs. Please note that the fixation leads to the loss of mScarlet-I fluorescence (compare Extended Data Fig. 7f). Dissociated cells from in utero electroporated brains were sorted for GFP (Neurog2) or RFP (MPRA). After sorting, cells were either directly used for nucleotide isolation or fixed, quenched, pelleted, flash frozen and stored at −80 °C as described above.

### RNA extraction, real-time quantitative PCR and RNA-seq library preparation

RNA from fixed or non-fixed cells was extracted using the Quick-RNA FFPE Miniprep kit (Zymo Research) or TRIzol (Thermo Fisher), respectively.

For qPCR, reverse transcription was performed using the Maxima H Minus Reverse Transcriptase (Thermo Fisher) with OligodT primer (Thermo Fisher) according to the instructions manual. Transcripts were quantified by using either the LightCycler 480 SYBR Green I Master Mix (Roche) or the Luna Universal qPCR Master Mix (New England Biolabs) with the appropriate primers (Supplementary Data 10) on a Roche LightCycler 480.

RNA-seq libraries were generated using the NEBNext Single Cell/Low Input RNA Library Prep Kit (New England Biolabs) according the manufacturer’s instruction.

### Methyl-HiC and in situ Hi-C

For Methyl-HiC and the low-input in situ Hi-C, we adapted current protocols^7,8^ (detailed experimental procedures can be found at https://www.protocols.io/view/methylhic-bif2kbqe/ and https://www.protocols.io/view/in-situ-hi-c-brd4m28w/, respectively)

Briefly, frozen pellets of fixed cells were thawed on ice, lysed with 0.2% Igepal-CA630 (Sigma-Aldrich), permeabilized with 0.5% SDS (Invitrogen) and digested with DpnII (New England Biolabs) overnight at 37 °C. Subsequently, sticky ends were filled with biotin-14-dATP (Life Technologies) followed by T4 Ligase (New England Biolabs)-based proximity ligation for at least 6 h at 16 °C. Thereafter, nuclei were lysed, reverse crosslinked overnight at 68 °C, purified by ethanol precipitation and sheared to ~550-bp DNA fragments using a Covaris S220 sonicator.

For in situ Hi-C, biotin pulldown was performed by incubation of the sheared DNA with MyOne Streptavidin T1 beads (Thermo Fisher) for 30 min at RT followed by on-bead biotin removal and end repair by incubating the samples for 30 min at RT in a reacting mix consisting of T4 DNA Polymerase, T4 Polynucleotide Kinase and DNA Polymerase I, Large (Klenow) Fragment (New England Biolabs). Thereafter, A-tailing using Klenow Fragment exo-minus (New England Biolabs) was performed for 30 min at 37 °C followed by ligation of NextFlex DNA barcodes (Perkin Elmer). Between each of the incubation steps the samples bound to the streptavidin beads were washed twice with washing buffer containing 0.05% Tween-20 (Sigma-Aldrich) and once with the respective buffer of the following reaction. Libraries were amplified on the streptavidin beads using the NEBNext Ultra Q5 II Master mix (New England Biolabs) using the following program: 98 °C for 30 s; (98 °C 10 s, 65 °C 75 s) × 10; 65 °C for 5 min; hold at 10 °C. After the amplification, the streptavidin beads were pelleted and the supernatant was purified twice using 0.7× AMPure XP beads (Agencourt) to reach an average fragment size of approximately 500 bp.

In the case of Methyl-HiC, end repair was performed by incubating the sonicated DNA with T4 DNA Polymerase (New England Biolabs) for 4 h at 20 °C followed by bisulfite conversion using the EZ DNA Methylation-Gold kit (Zymo Research) with the prior spike-in of methylation controls (~0.01%). For library construction, the Accel-NGS Methyl-Seq DNA Library kit (Swift Bioscience) was used according to the manufacturer’s instructions until the adaptor ligation step. After this step, biotin pulldown was performed using MyOne Streptavidin T1 beads (Thermo Fisher) followed by five washes with washing buffer containing 0.05% Tween-20 (Sigma-Aldrich) and two additional washes with low-TE water. Libraries were amplified on the streptavidin beads using the EpiMark Hot Start Taq (New England Biolabs) with Methyl-Seq Indexing Primers (Swift Bioscience) using following program: 95 °C 30 s; (95 °C 15 s, 61 °C 30 s, 68 °C 60 s) × 14; 68 °C 5 min; hold at 10 °C. Streptavidin T1 beads were pelleted and the prepared libraries within the supernatant were purified using 0.6× AMPure XP beads (Agencourt).

### The nucleosome occupancy and methylome sequencing assay

For the nucleosome occupancy and methylome sequencing (NOMe-seq) assay, we adapted current protocols^34,56^. A detailed experimental procedure can be found at https://www.protocols.io/view/nome-seq-of-fixed-cells-brdwm27e. Briefly, frozen pellets of fixed cells were thawed on ice, lysed with 0.2% Igepal-CA630 (Sigma-Aldrich) and incubated with M.CviPI (New England Biolabs) for 3 h at 37 °C with additional substitution of enzyme every hour. Thereafter, nuclei were lysed, reverse crosslinked overnight at 68 °C, purified by ethanol precipitation and sheared to ~550-bp fragments using a Covaris S220 sonicator. Methylation controls consisting of M.CviPI treated and sheared fully methylated pUC19 (Zymo Research) and unmethylated lambda DNA (Promega) were added to the samples (~0.05%) followed by bisulfite conversion using the EZ DNA Methylation-Gold kit (Zymo Research). For library construction, the Accel-NGS Methyl-Seq DNA Library kit (Swift Bioscience) was used according to the manufacturer’s instructions until the final library amplification step. Library amplification was performed in five separate reactions using the EpiMark Hot Start Taq (New England Biolabs) with Methyl-Seq Indexing Primers (Swift Bioscience) using the following PCR program: 95 °C for 30 s (95 °C for 15 s, 61 °C for 30 s and 68 °C for 60 s) × 11; 68 °C for 5 min, and hold at 10 °C. PCR reactions were pooled and purified using 0.8× AMPure XP beads (Agencourt).

### MPRA and CREs barcode association library generation

A detailed protocol for the MPRA library preparations can be found at https://www.protocols.io/view/mpra-library-preparation-bxdtpi6n/.

Briefly, RNA and DNA from fixed or unfixed cells were extracted using the Quick-DNA/RNA Microprep Plus Kit (Zymo Research) according to the corresponding manufacturer’s instructions. Purified RNA was treated with TURBO DNase (Thermo Fisher) and reverse transcribed with Maxima H Minus RT (Thermo Fisher) using Oligo(dT)18 Primer (Thermo Fisher). cDNA was purified with 1.5× AMPure XP magnetic beads (Agencourt). For both the DNA and cDNA libraries, unique molecular identifiers (UMIs) were added by PCR (98 °C for 30 s (98 °C for 10 s, 65 °C for 30 s, 72 °C for 1 min) × 3, 72 °C for 3 min, and hold at 4 °C) using the primers RV_univ_MPRA and FWD_mScar_Tn7_10UMI_3 (0.5 µM each). P7 and P5 sequencing adaptors were attached in two separate PCRs (98 °C for 30 s (98 °C for 10 s, 65 °C for 90 s) × (10 + X) 72 °C 5 min, and hold at 4 °C) using indexed Ad2.X^57^ and P5NEXTPT5 primers (0.1 µM each). Required PCR cycle numbers were estimated by qPCR and using one-tenth of the second PCR product as input. All PCRs were performed in 1× NEBNext Ultra II Q5 Master Mix (New England Biolabs), and PCR products were purified with 0.8× to 1.2× of AMPure XP magnetic beads. Final libraries were quantified by Qubit (Thermo Fisher) and Bioanalyzer 2100 (Agilent).

For the CREs barcode association library, 5 ng of the plasmid pool without minimal promoter and mScarlet-I was used to attach P5 and P7 sequencing adaptors in two separated PCRs. Both PCRs were performed using the NEBNext Ultra II Q5 Master Mix (New England Biolabs) with RV_univ_MPRA + FWD_CRS_Tn7 (0.5 µM each; PCR conditions: (98 °C for 30 s, (98 °C for 10 s, 65 °C for 30 s and 72 °C for 3 min) × 3; 72 °C for 3 min, and hold at 4 °C) and P5NEXTPT5 + indexed Ad2.X (0.1 µM each; PCR conditions: 98 °C for 30 s, (98 °C for 10 s, 65 °C for 90 s) × 10, 72 °C for 5 min, and hold at 4 °C), respectively. All primers used are listed in Supplementary Data 10.

### Library quality control and sequencing

Before sequencing, libraries were quantified by qPCR using either the NEBNext Library Quant kit (New England Biolabs, E7630S) or the KAPA Library Quantification Kit (scRNA-seq libraries only; Roche, 07960298001). Size distribution of the obtained libraries was assessed using the Agilent 2100 Bioanalyzer. Sequencing depths of the libraries are listed in Supplementary Data 1.

### scRNA-seq processing

Raw sequencing data were converted to fastqs using cellranger mkfastq (10x Genomics, v3.1.0). scRNA reads were aligned to the GRCm38 reference genome (mm10) and quantified using cellranger count (10x Genomics, v3.1.0).

### scRNA-seq quality control

We removed low-informative cells by filtering cells with less than 1,000 genes or 2,500 UMIs per cell detected. To lower doublet representation, we filtered cells with more than 7,000 genes per cell detected and the top 4% cells (estimated doublet percentage) with the highest number of UMIs. Finally, to remove any potential dead cells, we filtered cells that have more than 10% mitochondrial counts.

### scRNA-seq clustering and dimensionality reduction

Seurat (v3.1.5)^58^ was used to further process the cells passing the quality-control (QC) filters. After log transformation, feature selection using variance stabilizing transformation (top 2,000 most highly variable genes) and linear transformation, principal-component analysis was performed using the first 20 dimensions. After dimensionality reduction, Harmony^59^ was used to correct the batch effect between the two biological replicates. Next, we applied Louvain clustering (resolution = 0.3, n.start = 100, n.iter = 500) and visualized the data using UMAP (min.dist = 0.5, spread = 1, n.epochs = 500). As they represent very few cells, the microglia and mural clusters were further manually identified based on the UMAP projection and the subclustering of the NSC cluster. Cluster identify was determined based on the top 40 differentially expressed genes (MAST^60^, minimum log fold change of 0.25 and expressed in at least 25% of the cells in the cluster)

### scRNA-seq velocity and pseudotime analysis

The percentage of spliced and unspliced reads was calculated using Velocyto (v0.17)^61^ and RNA velocity was calculated using scVelo (dynamical model)^17^. Only cells passing the previously described QC were used and UMAP coordinates were transferred from Seurat. To calculate trajectory and pseudotime, we used Monocle3 (ref. ^18^), while retaining cluster assignment and UMAP coordinates from Seurat. A trajectory graph was constructed using the following parameters: minimal_branch_len = 20, ncenter = 600, geodesic_distance_ratio = 0.275. Cells belonging to the NSC cluster were assigned as root cells. To calculate the change of gene expression as a function of the pseudotime, we fitted a generalized additive model using cubic regression splines and REML smoothing for each of the top 3,000 most variable genes (expressed in at least 20 cells). The values were then rescaled per gene from 0 to 1.

### scATAC–seq processing

Raw sequencing data were converted to fastqs using cellranger-atac mkfastq (10x Genomics, v1.2.0). Reads were aligned to the GRCm38 reference genome (mm10) and quantified using cellranger-atac count (10x Genomics, v1.2.0) using integrated doublet removal.

### scATAC–seq quality control

We calculated the QC statistics separately for each replicate and filtered the combined 10× object (merged using cellranger --atac --aggr with no normalization). To ensure sufficient sequencing depth and high signal-to-noise ratio, we filtered cells with less than 10,000 unique fragments per cell and a TSS enrichment ratio of less than 8 or more than 25 (Extended Data Fig. 7f). To account for any remaining doublets after the automatic cellranger-atac filtering, we additionally removed cells with more than 120,000 unique fragments per cell. TSS enrichment was calculated as described previously^28^. In brief, Tn5 insertions located within ±2,000 bp relative from each TSS (strand corrected) were aggregated per TSS, normalized to the mean accessibility ±1,900–2,000 bp from the TSS and smoothed every 51 bp. The maximum smoothed value was reported as TSS enrichment. Fragment size distribution for cells passing the QC filters was calculated using ArchR^62^ and plotted with ggplot2.

### scATAC–seq clustering and dimensionality reduction

To obtain a set of initial clusters, we first counted the number of unique fragments in 5-kb genomic bins^63^. After binarization, the top 20,000 accessible windows were kept and the matrix was transformed using log term frequency-inverse document frequency (TF-IDF) transformation using Signac^63^. The normalized matrix was then used as an input for partial singular value decomposition (SVD) using irlba. After dimensionality reduction, Harmony^59^ was used to correct the batch effect between the two biological replicates. Next, we retained the first 20 dimensions, applied Louvain clustering with the following parameters: resolution = 1, n.start = 50 and n.iter = 50, and visualized the data using UMAP (min.dist = 0.5, spread = 1.5, n.epochs = 2,000).

For each cluster, peak calling was performed on the Tn5-corrected insertions as described in Granja et al. (2019)^28^. Peak size was then normalized to 501 bp in length, filtered by the mm10 ENCODE blacklist and then peaks were merged into a union set as previously described^28^.

Next, this high-quality peak set was used to generate the final clustering and visualization. First, fragments contained within peaks were calculated using Signac, binarized, and the top 25,000 variable peaks were identified (using aggregated counts per million from the initial bin-based clusters). The count matrix associated with those peaks was then again subjected to TF-IDF normalization followed by SVD as described above. After batch correction using Harmony^59^, the first 20 dimensions were retained and clusters were identified using Louvain algorithm (resolution = 0.3, n.start = 100, n.iter = 200). Data were visualized using UMAP embedding (min.dist = 0.5, spread = 1.5, n.epochs = 2,000). Cluster identity was determined based on the top 40 differentially accessible gene bodies (Student’s *t*-test, minimum log fold change of 0.25 and expressed in at least 25% of the cells in the cluster).

### scATAC–seq calculation of promoter and gene body

To calculate gene body accessibility scores, we counted the number of unique fragments along the whole span (TSS – transcription termination site of protein-coding genes (EnsDb.Mmusculus.v79), extended 2,000 upstream of TSS. To calculated promoter scores, we counted the number of unique fragments along promoters of protein-coding genes (defined as the sequence −2,000 bp to +200 bp of the TSS).

### scATAC–seq motif and ChIP–seq accessibility deviations

Motif accessibility was calculated using chromVAR^24^ as implemented in the Signac^63^. In brief, position-weight matrices were obtained from the JASPAR 2020 motif database^64^, to which entries present in the JASPAR 2018 version but subsequently removed, were manually added. Each accessibility peak was then tested for the presence/absence of each TF motif and GC bias-corrected deviations were computed using the chromVAR ‘deviations’ function as implemented in Signac (‘RunChromVar’). Accessibility deviations associated with ChIP–seq peaks were computed analogously, but the overlap of ChIP–seq peaks and scATAC peaks was used as entry to chromVAR instead.

### scATAC–seq unique peaks identification

Cluster-specific peaks were identified using feature binarization as described^28^. In brief, pseudobulk replicates were created for clusters with *N* cells < 100, while real biological replicates were used for the remaining clusters. Peaks were considered as unique if they had an adjusted *P* value less than 0.01 and minimum log fold change of 0.25 to the next highest cluster. The identified unique peaks (Supplementary Data 3) were split into two categories: promoter associated (less than 500 bp away from a TSS) and distal (more than ±5 kb away from an annotated TSS).

### Transcription factor footprinting

The Tn5-normalized accessibility around TF motifs was calculated as previously described^65^ using the ArchR package^62^. The expected Tn5 bias was substracted from the calculated footprints to generate the final footprint plots^62^. The positions of the respective motifs within the ChIP–seq peaks were identified using the R package motifmatcher.

### Label transfer and co-embedding

To integrate scRNA and scATAC datasets, we used Seurat’s canonical correlation analysis^58^. In brief, we first identified transfer anchors using the top 5,000 most variable genes shared across both datasets using FindTransferAnchors (dims = 1:20, k.anchor = 20, k.filter = 200, k.score = 30, max.features = 500). We then transferred the scRNA-based labels using the inferred anchors and the harmony corrected low-dimensional coordinates as weight reduction. After we confirmed the high-confidence values of the prediction scores (Extended Data Fig. 7f), we then co-embedded the scRNA and the scATAC cells in the same low-dimensional space and recalculated UMAP embedding (Extended Data Fig. 7f; dims = 1:20, n.epochs = 2,000, spread = 1.5, min.dist = 0.5). To enable more robust downstream correlation-based analysis, we used the previously described Cicero-based *k*-nearest neighbor (kNN) approach to group scATAC–seq accessibility (4,892 groups, kNN = 50) match grouped gene expression (based on scRNA-seq closest neighbors).

### Identifying pairs of matched genes and predicted enhancers

To identify putative enhancers, where the accessibility of the predicted distal regions correlated with changes in gene expression (and not accessibility of the promoter), we adapted the approach first described by Granja et al. (2019)^28^. Briefly, the correlation between the log-normalized matched grouped scATAC and scRNA values was calculated for each pair of distal scATAC peaks (at least 5 kb away from any annotated TSS) and gene promoters within a maximum genomic distance of 500 kb. The significance of the calculated correlations was determined using a *trans*-based null correlation and peak-to-gene links with significance of FDR < 0.1 and a Pearson correlation > 0.35 were considered as positively correlated (referred to as putative enhancer–gene pairs, or EGPs, for simplicity). In addition, we also identified two additional classes of peak-to-gene relationships: negatively correlated (FDR < 0.1 and *r* < −0.35) and control pairs (FDR > 0.1 and −0.35 < *r* < 0.35). As the number of control pairs was much higher than either positively or negatively correlated EGPs, we subsampled this category to match the positively correlated pairs (*n* = 16,978). Cluster-specific pairs were determined using pseudobulk feature binarization as described above.

### Integrated pseudotime analysis

Pseudotime on the combined integrated scRNA–scATAC object was calculated using Monocle3, analogous to scRNA-seq alone. Imputed gene expression values based on gene body accessibility and integration vectors as described previously were used together with measured scRNA values to construct a cellDataSet object, retaining cluster assignment (scRNA based) and UMAP coordinates from Seurat. To calculate the change of accessibility and motifs deviations as a function of the pseudotime, we fitted a generalized additive model using cubic regression splines and REML smoothing, analogous to gene expression for scRNA-seq data. The values were then rescaled per gene from 0 to 1. For each EGP, we then ordered the cells based on their pseudotime and calculated the pseudotime difference between their respective maxima, which was used to infer the relationship between enhancer accessibility and gene expression (Fig. 3). An analogous process was repeated for TF motif deviations and the correlation between the gene expression pattern of each TF and its corresponding motif accessibility along the pseudotime was computed as well.

### MPRA data processing

In total, 50-bp paired-end (PE) reads from the CRE barcode association were trimmed using cutadapt with the following parameters: --m 12 --a GAATTCATCTGGTA --G GACCGGATCAACT --u 1 discard-untrimmed. Two 150-bp PE reads were first filtered using cutadapt (--m 12 --a GAATTCATCTGGTACCTCGGTTCACGCAATG --G CCAGGACCGGATCAACT --u 1 --discard-untrimmed --action = none --interleaved | cutadapt --g GAATTCATCTGGTACCTCGGTTCACGCAATG --G CCAGGACCGGATCAACT --discard-untrimmed --action = none --interleaved) and, subsequently, forward and reverse reads were trimmed individually using --l 12 for the barcode, --g CCAGGACCGGATCAACT --discard-untrimmed (forward CRE reads) or --g GAATTCATCTGGTACCTCGGTTCACGCAATG --discard-untrimmed (reverse CRE read). Trimmed fastq reads for both 50-bp PE and 150-bp PE reads were separated based on a 5′ 4-bp identifier and CRE barcode association was performed separately on WT and mutant sequences using the MPRAflow association pipeline^66^. The resulting pickle libraries were merged to increase the number of recovered CREs and filtered for 50-bp PE reads from the DNA or RNA libraries were trimmed using cutadapt with the following parameters: --u 1 --a GAATTCTCATTAC --A TCGACCGCAAGTTGG --discard-untrimmed. Count tables for RNA and DNA reads were generated using MPRAflow (--bc-length 12 and --mpranalyze) and subsequently imported into MPRAnalyse^30^ to calculate the MPRA signal, which represents either alpha.score or mad.score for pooled or cell-type-specific MPRA libraries, respectively. Statistically significant active enhancers are defined by a mad.score *P* value ≤ 0.05. For comparison of replicates the normalized DNA/RNA read counts as well as ratio of sums were calculated as previously described^67^. Motif enrichment was calculated using MonaLisa^68^.

### Hi-C mapping and quality control

We mapped the joint Hi-C/DNA methylation data to the mm10 genome using JuiceMe^69^. In situ Hi-C data were mapped using Juicer. Only uniquely mapping reads (mapq > 30) were retained for further analysis. After removal of PCR duplicates, reads were translated into a pair of fragment ends (fends) by associating each read with its downstream fend. CpG methylation was assessed using MethylDackel (https://github.com/dpryan79/MethylDackel/), in a ‘mergeContext’ mode with the first six nucleotides omitted from further analysis. Reads from individual replicates were pooled and only Cs in a CpG context with at least 10× total coverage were further analyzed. For Hi-C, reads mapping to the same restriction fragment or separated by less than 1 kb were excluded from further analysis. The QC metrics are reported in Supplementary Data 1.

### NOMe-seq mapping and quality control

NOMe-seq reads were first trimmed using trim galore in a PE mode with --clip_R1 = 1 and --clip_R2 = 15 to account for the A-tail deposited by adaptase. Reads were then mapped using bismark in PE mode, deduplicated and methylation calls were extracted (--ignore 6 and --CX). Coverage files were then produced using coverage2cytosine in --nome-seq mode. Only calls with at least 5× coverage per replicate or 10× coverage in the merged data were used for the subsequent analysis. Pearson correlation of CpG methylation in 1-kb bins displays a correlation of ≥0.77 between replicates.

### Quality control of bisulfite conversion efficiency

To determine the efficiency of the bisulfite conversion, we determined the proportion of CpG methylation that was detected in fragments mapping to the unmethylated lambda DNA sequence. For Methyl-HiC (Extended Data Fig. 7f) and NOMe-seq, this represents ~0.5% or ~3.0, suggesting a conversion rate of 99.5% or 97%, respectively. To calculate the detection rate for the Methyl-HiC, we determined the proportion of CpG on fully methylated pUC19 plasmid DNA and observed >96.5%, suggesting a false negative rate of less than 3.5% (Extended Data Fig. 7f). Furthermore, we detected a high Pearson correlation of CpG methylation levels in 1-kb bins across replicates for both Methyl-HiC (*r* ≥ 0.93) and NOMe-seq (*r* ≥ 0.87).

### Hi-C data processing

The filtered fend-transformed read pairs were converted into ‘misha’ tracks and imported into the genomic mm10 database. They were normalized using the Shaman package (https://tanaylab.bitbucket.io/shaman/index.html) and the Hi-C score was calculated using a kNN strategy on the pooled replicates as previously described^15^ with a kNN of 100.

### Contact probability, insulation and TAD boundary calling

Contact probability as a function of the genomic distance was calculated as previously described^15^.

To define insulation based on observed contacts, we used the insulation score as previously defined^15,70^. The insulation score was computed on the pooled contact map at 1-kb resolution within a region of ±250 kb and was multiplied by (−1) so that a high insulation score represents strong insulation. Domain boundaries were then defined as the local 2-kb maxima in regions where the insulation score is above the 90% quantile of the genome-wide distribution. Differential TAD boundaries were identified as previously described^15^ using genome-wide normalized insulation scores.

### Compartments and compartment strength

We first calculated the dominant eigenvector of the contact matrices binned at 250 kb as described previously^71^ using scripts available at https://github.com/dekkerlab/cworld‐dekker/. To determine the compartment strength, we plotted the log_2_ ratio of observed versus expected contacts (intrachromosomal separated by at least 10 Mb) either between domains of the same (A–A, B–B) or different type (A–B), as previously described^15^. We calculated compartment strength as the ratio between the sum of observed contacts within the A and B compartments and the sum of intercompartment contacts (AA + BB)/(AB + BA).

### Average topologically associated domain contact enrichment

The insulation and contact enrichment within TADs was calculated as previously described^15^. Briefly, TAD coordinates were extended upstream and downstream by the TAD length and this distance was split into 100 equal bins. The observed versus expected enrichment ratio was calculated in each resulting 100 × 100 grid (per TAD) and the average enrichment was plotted per bin. Average CpG DNA methylation was calculated for each of these 100 bins per TAD and was represented as the mean ± 0.25 quantiles.

### Aggregated and individual contact strength at pairs of genomic features

To calculate the contact enrichment ratio at pairs of genomic features (such as ChIP–seq peaks, accessible motif sites or linked pairs of enhancers and promoters), we used two complementary approaches. First, we aggregated Hi-C maps to calculate the log_2_ ratio of the observed versus expected contacts within a window of a specific size, centered on the pair of interest, as described previously^15^. Furthermore, we calculated the average enrichment ratio of the contact strength in the center of the window (central nine bins) versus each of the corners. This analysis is useful to identify general patterns of changes in chromatin interactions in the data, but cannot distinguish the heterogeneity and the contribution of individual pairs. To address this question, we also extracted the kNN-based Hi-C score in a 10-kb window centered around each of the pairs separately and represented the data as a scatterplot or box plot. Significance was then calculated using the Wilcoxon rank test.

### Average enrichment of linear marks at genomic features

We used SeqPlots^72^ to calculate the average enrichment of linear chromatin marks (DNA methylation, chromatin accessibility, ChIP–seq) in window centered around the genomic feature of interest, or along scaled gene bodies.

### Inferring Hi-C contact strength and CpG DNA methylation associated with transcription factor binding motifs

Although ChIP–seq (and related techniques) remains the method of choice to identify the real binding sites of a TF, in many cases this is not feasible due to the lack of suitable antibodies. We reasoned that we could use predicted TF motifs at highly accessible peaks to examine how such potentially occupied motifs are spatially positioned to each other and how they are associated with changes in DNA methylation. Briefly, for each TF motif, we first identified the scATAC-based peaks that contain the predicted binding motif and then ranked these sites based on their maximum accessibility in aggregated pseudobulk scATAC-based clusters. To not over-penalize rare motifs, we selected the top 5,000 most highly accessible regions per motif and created point-based regions centered at the corresponding motif. TFs (and their corresponding motifs) that were not expressed in our data (reads per kilobase per million (RPKM) > 1 based on pseudobulk scRNA-seq data) or were not within the top 3,000 most variable genes (based on scRNA-seq) were discarded from further analysis.

To create a set of control regions, not enriched for any particular motif, we first selected a set of the top 50,000 most highly accessible sites, analogous to motif-based analysis. Then, we sampled 5,000 peaks randomly 1,000 times to create a set of highly robust background peaks with similar characteristics. Next, we created pairs of these regions separated by at least 10 kb, filtered for intraTAD interactions and extracted the maximum Hi-C score (for each cell type) within a square window of 10 × 10 kb centered on each pair. We then calculated the median value per TF motif for each cell type and repeated this for each of the 1,000 controls to generate a background distribution. To normalize for any non-specific interaction, we calculated the mean of the 1,000 controls per cell type and subtracted it from the TF motif values. We then calculated the maximum Hi-C score (from all three cell types) and the standard deviation per motif and plotted them as a scatterplot using ggplot2. To calculate the statistical significance, we used permutation-based analysis (utilizing the 1,000 repeated sampling of the random regions) and considered motifs with *P* < 0.05 as significant. We also depicted the Pearson’s correlation between the motif deviation scores and the expression of the matching TF as described for Fig. 3e.

For the analysis of DNA methylation, we extracted the average CpG methylation in 500-bp windows centered on the motif-based regions described above. We then generated a random permutation-based background exactly as described for the Hi-C and plotted the minimum DNA methylation and standard deviation for the three cell types as a scatterplot. Statistical significance was calculated as described above.

As a complementary analysis, we calculated the average Hi-C score and enhancer DNA methylation for positively correlated EGPs and presented the data as scaled median Hi-C score per TF motif.

### External datasets

The following external datasets were used: Sox2 ChIP–seq (GSE35496)^73^, Pax6 ChIP–seq (GSE66961)^74^, Neurog2 ChIP–seq (GSE63621)^75^, Eomes ChIP–seq (GSE63621)^75^, Neurod2 ChIP–seq (GSE67539)^76^ and Tbr1 ChIP–seq (GSE71384)^77^. The ChIP–seq datasets were uniformly processed using the ENCODE ChIP–seq pipeline (https://github.com/ENCODE-DCC/chip-seq-pipeline2/). Publicly available 10× multiome data from the E18 mouse brain were obtained from https://support.10xgenomics.com/single-cell-multiome-atac-gex/datasets/2.0.0/e18_mouse_brain_fresh_5k/ and reanalyzed as described above.

### Sample sizes

No statistical methods were used to predetermine sample sizes but our sample sizes are similar to those reported in previous publications^3,4,8,15,34^.

### Reporting Summary

Further information on research design is available in the Nature Research Reporting Summary linked to this article.

## Online content

Any methods, additional references, Nature Research reporting summaries, source data, extended data, supplementary information, acknowledgements, peer review information; details of author contributions and competing interests; and statements of data and code availability are available at 10.1038/s41593-021-01002-4.

## Supplementary information

Reporting Summary

Supplementary Data 1 Assay-specific quality matrices for scRNA-seq, scATAC–seq, Methyl-HiC, Hi-C, NOMe-seq, RNA-seq and MPRA.

Supplementary Data 2 Gene expression levels across the identified scRNA-seq clusters. Related to Fig. 1g.

Supplementary Data 3 Accessibility peaks identified in each scATAC–seq cluster as well as the generate union peakset.

Supplementary Data 4 Cell-type-specific transcription factor motif accessibility and the expression levels of the corresponding factor. Related to Fig. 2c.

Supplementary Data 5 Cell-type-specific accessibility levels and annotation of the union peakset. Related to Fig. 2g.

Supplementary Data 6 Genomic location, cell-type-specific Hi-C scores as well as methylation levels of identified enhancer–gene pairs. Related to Figs. 3a, 6a and 6e.

Supplementary Data 7 Pseudotemporal relationship between positively correlated enhancer–gene pairs. Related to Fig. 3d.

Supplementary Data 8 Genomic coordinates and cell-type-specific insulation scores of the identified TADs. Related to Fig. 5d.

Supplementary Data 9 Cell-type specificity and variance of chromatin interactions as well as DNA methylation levels at transcription factor motifs. Related to Fig. 7a,e.

Supplementary Data 10 List of primers used for cloning, MPRA and qPCR.

## Data Availability

All raw and processed sequencing data are available in the Gene Expression Omnibus repository under accession code GSE155677. An interactive version of the single-cell and the genomics data can be visualized at https://shiny.bonevlab.com/.

## Code availability

The code used for generating the data and all the figures are freely available under https://github.com/BonevLab/Noack_et_al_NatNeuro2021/. Methyl-HiC data were processed using the JuiceMe pipeline, available at https://github.com/aidenlab/JuiceMe/. The R package to compute the expected tracks and the Hi-C scores is available at https://bitbucket.org/tanaylab/shaman/.
