# Supplementary material for: Multimodal profiling of the transcriptional regulatory landscape of the developing mouse cortex identifies Neurog2 as a key epigenome remodeler
Source: Nat Neurosci. 2022 Feb 7;25(2):154–67. doi: 10.1038/s41593-021-01002-4 (PMC8825286; doi:10.1038/s41593-021-01002-4)
Supplement: Supplementary file 1 — Reporting Summary [file 41593_2021_1002_MOESM1_ESM.pdf]

nature research

Corresponding author(s): Boyan Bonev

Last updated by author(s): Dec 7, 2021

Reporting Summary

Nature Research wishes to improve the reproducibility of the work that we publish. This form provides structure for consistency and transparency in reporting. For further information on Nature Research policies, see our Editorial Policies and the Editorial Policy Checklist.

Statistics

For all statistical analyses, confirm that the following items are present in the figure legend, table legend, main text, or Methods section.

n/a	Confirmed	
☐	☒	The exact sample size (*n*) for each experimental group/condition, given as a discrete number and unit of measurement
☐	☒	A statement on whether measurements were taken from distinct samples or whether the same sample was measured repeatedly
☐	☒	The statistical test(s) used AND whether they are one- or two-sided<br>*Only common tests should be described solely by name; describe more complex techniques in the Methods section.*
☐	☒	A description of all covariates tested
☐	☒	A description of any assumptions or corrections, such as tests of normality and adjustment for multiple comparisons
☐	☒	A full description of the statistical parameters including central tendency (e.g. means) or other basic estimates (e.g. regression coefficient) AND variation (e.g. standard deviation) or associated estimates of uncertainty (e.g. confidence intervals)
☐	☒	For null hypothesis testing, the test statistic (e.g. *F*, *t*, *r*) with confidence intervals, effect sizes, degrees of freedom and *P* value noted<br>*Give P values as exact values whenever suitable.*
☒	☐	For Bayesian analysis, information on the choice of priors and Markov chain Monte Carlo settings
☒	☐	For hierarchical and complex designs, identification of the appropriate level for tests and full reporting of outcomes
☐	☒	Estimates of effect sizes (e.g. Cohen's *d*, Pearson's *r*), indicating how they were calculated

Our web collection on statistics for biologists contains articles on many of the points above.

Software and code

Policy information about availability of computer code

Data collection	No software was used for data collection.
Data analysis	imageJ (1.53c), FlowJo (10.8.0), cellranger (v3.1.0), cellranger-atac (v1.2.0), Seurat (v3.1.5), Harmony (v0.1), MAST, Velocyto (v0.17), scVelo, Monocle3, ArchR, Signac, chromvar, JuiceMe, MethylDackel, Shaman package, Cworld, SeqPlots, R. All code as well as a list of all used packages including version numbers is available at https://github.com/BonevLab/Noack_et_al_NatNeuro2021.

For manuscripts utilizing custom algorithms or software that are central to the research but not yet described in published literature, software must be made available to editors and reviewers. We strongly encourage code deposition in a community repository (e.g. GitHub). See the Nature Research guidelines for submitting code & software for further information.

Data

Policy information about availability of data

All manuscripts must include a data availability statement. This statement should provide the following information, where applicable:

- Accession codes, unique identifiers, or web links for publicly available datasets
- A list of figures that have associated raw data
- A description of any restrictions on data availability

All raw and processed sequencing data are available in the Gene Expression Omnibus (GEO) repository: GSE155677. An interactive version of the single-cell and the genomics data can be visualized at https://shiny.bonevlab.com.

Field-specific reporting

Please select the one below that is the best fit for your research. If you are not sure, read the appropriate sections before making your selection.

☒ Life sciences ☐ Behavioural & social sciences ☐ Ecological, evolutionary & environmental sciences

For a reference copy of the document with all sections, see nature.com/documents/nr-reporting-summary-flat.pdf

Life sciences study design

All studies must disclose on these points even when the disclosure is negative.

Sample size	Sample sizes for all data types are provided in the Supplementary Data Table 2. Sample sizes for scRNA-seq/scATAC-seq were chosen based upon the ability to get representative data described based upon numerous studies in the field. Sample size for MPRA and methylHiC/HiC/NOMe-seq to ensure replication of the results with affordable cost.
Data exclusions	No samples were excluded from the analysis. For scRNA and scATAC, cells with low quality control (QC) values were excluded from the final analysis as described in the methods section.
Replication	For scRNA/scATAC/MPRA two and for methylHiC three biological replicates were used. For Neurog2 functional experiments the following biological replicates were used: 3x for Hi-C, 2x for NOMe-seq, 2x RNA-seq. Unless otherwise stated, all other experiments were performed in biological triplicates. All attempts of replications were successful.
Randomization	For all experiments involving mice, embryos from one litter were randomly assigned to the experimental group. For the scRNA, scATAC, MPRA and Methyl-HiC experiments, there was no randomization performed as they do not involve multiple study groups. For the in utero electroporation experiments animals were assigned to the control (GFP) or experimental (Neurog2) group based on the construct used during the procedure.
Blinding	The authors were not blinded to the group during the data collection and analysis.

Reporting for specific materials, systems and methods

We require information from authors about some types of materials, experimental systems and methods used in many studies. Here, indicate whether each material, system or method listed is relevant to your study. If you are not sure if a list item applies to your research, read the appropriate section before selecting a response.

Materials & experimental systems

n/a	Involved in the study	
☐	☒	Antibodies
☒	☐	Eukaryotic cell lines
☒	☐	Palaeontology and archaeology
☐	☒	Animals and other organisms
☒	☐	Human research participants
☒	☐	Clinical data
☒	☐	Dual use research of concern

Methods

n/a	Involved in the study	
☒	☐	ChIP-seq
☐	☒	Flow cytometry
☒	☐	MRI-based neuroimaging

Antibodies

Antibodies used	Pax6, mouse, Alexa Fluor 488 (BD Bioscience, Cat. N: 561552); Eomes, mouse, PE (BD Bioscience,Cat. N: 566749); anti-β-Tubulin Class III, mouse, Alexa Fluor 647 (BD Bioscience,Cat. N: 560394); anti-GFP, chicken (Abcam, Cat. N: ab13970); anti-Neurog2, rabbit (Cell Signalling, Cat. N: 13144). Antibodies were used both for immunohistochemistry and flow-cytometry.
Validation	Antibodies were validated by by the corresponding manufacturer:<br>Pax6: (https://www.bdbiosciences.com/us/applications/research/intracellular-flow/intracellular-antibodies-and-isotype-controls/anti-human-antibodies/pe-mouse-anti-human-pax-6-o18-1330/p/561552)<br>Eomes: https://www.bdbiosciences.com/eu/reagents/research/antibodies-buffers/immunology-reagents/anti-human-antibodies/cell-surface-antigens/pe-mouse-anti-eomes-x4-83/p/566749<br>Tubb3: https://www.bdbiosciences.com/us/reagents/research/antibodies-buffers/cell-biology-reagents/cell-biology-antibodies/alexa-fluor-647-mouse-anti--tubulin-class-iii-tuj1/p/560394<br>Neurog2 (https://www.cellsignal.com/products/primary-antibodies/neurogenin-2-d2r3d-rabbit-mab/13144)<br>GFP (https://www.abcam.com/gfp-antibody-ab13970.html)<br>Additionally, the specificity of antibodies for ImmunoFACS (Pax6, Eomes, Tubb3) were confirmed by qPCR (Extended Data Fig. 7b).

Animals and other organisms

Policy information about studies involving animals; ARRIVE guidelines recommended for reporting animal research

Laboratory animals	Time-mated pregnant C57BL/6JRj female mice (embryonic day E14). Mice were kept under standard housing conditions (room temperature 22C, humidity 55%) according to local regulations of the Regierung Oberbayern, Germany. Mouse embryos were used sex independent.
Wild animals	No wild animals were used.
Field-collected samples	No field-collect samples were used.
Ethics oversight	Animal experiments were approved by the animal welfare commission of the Regierung Oberbayern, Germany under the animal license number: ROB-55.2-2532.Vet_02-19-175

Note that full information on the approval of the study protocol must also be provided in the manuscript.

Flow Cytometry

Plots

Confirm that:

☒ The axis labels state the marker and fluorochrome used (e.g. CD4-FITC).

☒ The axis scales are clearly visible. Include numbers along axes only for bottom left plot of group (a 'group' is an analysis of identical markers).

☒ All plots are contour plots with outliers or pseudocolor plots.

☒ A numerical value for number of cells or percentage (with statistics) is provided.

Methodology

Sample preparation	Dissected cortex was dissociated using a papain-based neural dissociation kit (Miltenyi Biotec, Cat. N: 130-092-628) according to the manufacturer protocol with minor modifications. Dissociated cells were fixed in 1% Formaldehyde, quenched with 0.2M Glycine followed by permeabilization using 0.1% Saponin (Sigma-Aldrich, Cat. N: SAE0073). Cells were stained for 1h at 4C for Pax6 (1:40; BD Bioscience, Cat. N: 561664), Eomes (1:33; BD Bioscience, Cat. N: 566749) and Tubb3 (1:14; BD Bioscience, Cat. N: 560394) in staining buffer containing 0.1 % Saponin. Stained cells were washed 4 times including one wash with washing buffer containing DAPI (1:1000; ThermoFisher, Cat. N: 62248). Cells were passed through a 40µM cell strainer and immediately FAC-sorted. A detailed protocol for the immunoFACS can be found at: https://www.protocols.io/private/AFB8DC003DF7C61EA1E62D534934FD23.
Instrument	FAC-sorting was performed on a BD FACSAria Fusion (BD Bioscience) with four lasers (405, 488, 561, 640) using a 100µm nozzle.
Software	BD FACSDiva
Cell population abundance	Abundance of relevant cell populations are shown in Extended Data Fig. 7a. Briefly, Pax6+ cells (7.67%), Eomes+ cells (11.19%), Tubb3+ cells (23.6%). Purity of FAC-sorted cells were determined by qPCR of relevant marker genes (Extended Data Fig. 7b).
Gating strategy	After selecting singlets using forward and side scatter, cells in G0G1 were identified by genomic content based on DAPI staining. Subsequently, these cells were divided into Tubb3 high for PN and low for progenitor cell types. The progenitor population was further subdivided into Pax6-high/Eomes-low for NSC and Eomes-high for IPC. The set gates are displayed in Extended Data Fig. 6n and 7a.

☒ Tick this box to confirm that a figure exemplifying the gating strategy is provided in the Supplementary Information.
